# Breast cancer genomic subtype-specific copy number alterations in metastases

**DOI:** 10.1038/s41598-026-60972-2

**Published:** 2026-07-11

**Authors:** Katherine Eason, Christopher Boursnell, Raquel Manzano, Paul A. W. Edwards, Suet-Feung Chin, Florian Markowetz, Oscar M. Rueda, Carlos Caldas

**Affiliations:** 1 Cancer Research UK Cambridge Institute, University of Cambridge, Cambridge, UK; 2 MRC Biostatistics Unit, University of Cambridge, Cambridge, UK; 3 Institute of Metabolic Science and Department of Clinical Biochemistry, University of Cambridge, Cambridge, UK; 4 Faculty of Medicine, Lautenberg Center for Immunology and Cancer Research, Institute of Medical Research Israel-Canada, Hebrew University of Jerusalem, Jerusalem, Israel

**Keywords:** Biomarkers, Cancer, Computational biology and bioinformatics, Genetics, Oncology

## Abstract

Breast cancer is the second most common cancer globally, with rising incidence and poor prognosis following recurrence. Genomic analysis of primary breast tumours has identified subtypes with widely varying risk of relapse, highlighting the importance of tumour genomics in understanding metastasis. However, the genomic alterations associated with metastatic transformation—and how they differ between genomic subtypes—remain unclear due to limited sample sizes, lack of primary tumour baselines, and limited genomic coverage by panel sequencing. To address this gap, we analysed nearly 1300 whole-genome sequenced unmatched primary tumours and metastases using a unified computational pipeline. Somatic copy number profiles were classified into genomic subtypes, called the Integrative Clusters, with an improved classifier. By employing various genome-wide approaches, we identify candidate genes in regions with copy number alterations enriched or depleted in metastases, and nominate biological pathways that may contribute to metastatic disease in each genomic subtype. These subtype-specific candidates provide a framework for prioritising therapeutic hypotheses and future functional studies in metastatic breast cancer.

## Introduction

Breast cancer is the second most common cancer diagnosis worldwide, and incidence is rising across much of Europe and the world^1^. Recurrence of breast cancer after initial treatment is common^2,3^, and the prognosis for patients after recurrence is very poor^4,5^.

Previously, our analysis of 2000 primary breast tumours defined ten genomic subtypes of breast cancer—which we call the Integrative Clusters—based on their somatic copy number alterations (CNAs)^6,7^. The molecular and clinical characteristics of these subtypes are summarised in Table 1. Where relevant, we distinguish ER-positive and ER-negative tumours classified as IntClust4 by CNA profile as IntClust4ER+ and IntClust4ER-, respectively. Later work confirmed that the genomic landscape of breast cancer is dominated by CNAs, as opposed to point mutations^8^. We later showed that these genomic subtypes are heterogeneous in their risk of relapse over time^5^. This demonstrates that the CNA landscape of breast tumours is key to understanding the factors that lead to relapse. However, the genomic events associated with the spread of metastatic cells from the primary breast tumour—a process called metastatic seeding—remain poorly understood^3,9^.

The genomes of breast metastases differ from those of primary tumours, but previous efforts to characterise these differences have been limited by lack of comparison to a primary tumour baseline^10^, small sample sizes^11^, or sequencing being restricted to known cancer-associated genes^12,13^. These genomic differences can be due to either the accumulation of further alterations after metastatic seeding, or clonal selection of cells in the primary with the highest fitness to metastasise, which then expand in the metastatic niche^13^. Alterations that are differentially enriched between primaries and metastases may therefore provide insight into candidate mechanisms associated with metastatic seeding.

Here, using two large cohorts totalling nearly 1300 whole-genome sequenced (WGS) unmatched primary tumours (ICGC) and metastases (Hartwig Medical Foundation) encompassing invasive breast cancers from all clinical subtypes (ER+, HER2+, and triple negative), we aim to describe the CNAs which are enriched or depleted (termed differential copy number) in metastases belonging to each Integrative Cluster (IntClust). Because these primary and metastatic samples were obtained from separate cohorts, these comparisons identify metastasis-associated differences in CNA frequency rather than longitudinal evolutionary changes within matched patients. The clinical characteristics of these two cohorts are summarised in Supplementary Table 1.

Sequencing data from these cohorts was re-processed using a unified computational pipeline to remove batch effects arising from software differences. CNA profiles were then generated using a consensus approach, and tumours classified into genomic subtypes using a recently-developed improved classifier^14^. Then, using both bin- and arm-level approaches, we identify a set of genes which fall in regions with differential copy number, and nominate candidate metastasis-associated mechanisms that may be linked to their alteration. By linking metastasis-associated CNAs to the genomic subtype in which they arise, this framework may help prioritise subtype-specific therapeutic hypotheses, candidate biomarkers, and future functional studies in metastatic breast cancer.

**Table 1:** Molecular and clinical characteristics of the Integrative Clusters. Fraction of genome altered, metastasis-associated CNAs and potential therapeutic opportunities reflect analyses and hypothesis-generating interpretations from the present study.

IntClust	Defining copy number features	Predominant clinical subtype	Clinical summary	Fraction of genome altered	Metastasis-associated CNAs	Potential therapeutic opportunities
IntClust1	17q23 amplification	ER+/HER2-	Intermediate prognosis; high grade	High	*RBM39*, *PHF20* gains	ER signalling; endocrine resistance
IntClust2	11q13/14 amplification	ER+/HER2-	Poor prognosis	High	Not identified	Not identified
IntClust3	1q gain; 16q loss	ER+/HER2-	Good prognosis; low grade; low lymph node involvement	Intermediate	HR/DDR losses; increased HRD	PARP/platinum/DDR targeting
IntClust4ER+	Absence of CNAs	ER+/HER2-	Good prognosis; intermediate grade	Low	*ERBB3*, MAPK/cell-cycle gains	HER3/MAPK; CDK4/6 targeting
IntClust4ER-	No defining CNAs	Triple negative	Intermediate prognosis; high grade	High	Not identified	Not identified
IntClust5	ERBB2 amplification	HER2+	Good prognosis with targeted therapy; high grade; high lymph node involvement; lower age	Low	*YES1* gain	HER2-therapy resistance; YES/SRC inhibition
IntClust6	8p12 amplification	ER+/HER2-	Intermediate prognosis	High	Not identified	Not identified
IntClust7	16p gain; 16q loss; 8q amplification	ER+/HER2-	Good prognosis; low grade; higher age	High	*KEAP1* loss; *MDM4* gain	Not identified
IntClust8	1q gain; 16q loss	ER+/HER2-	Good prognosis; low grade; higher age	Intermediate	*ERBB3*, *CDK4*, *MDM2* gains	HER3/MAPK; CDK4/6; MDM2-p53 axis
IntClust9	8q gain; 20q amplification	ER+/HER2-	Intermediate prognosis; high grade	Intermediate	*VRK1* gain	DNA-damaging therapy resistance
IntClust10	5q loss; 8q loss; 10p gain; 12p gain	Triple negative	Poor prognosis; high grade; lower age	High	*RPS6KB2*, *ADHFE1* gains	mTOR/S6K; metabolic stress

## Results

### Primary tumours harbour distinct copy number alterations in each genomic subtype

The landscape of CNAs in the ICGC cohort of primary tumours (*n* = 532; Supplementary Fig. 1) showed similar patterns of alterations as we and others have previously reported in primary lesions^6,12^. These included common subtype-agnostic events such as 1q, 8q and 16p gain, and 8p and 16q loss, as well as subtype-specific aberrations such as *ERBB2* amplification in the predominantly HER2+ IntClust5 (*n* = 54), 8p11-12 amplification in IntClust6 (*n* = 25), and 11q13-14 amplification in IntClust2 (*n* = 21).

Analysis of the timing of gains (Supplementary Fig. 2a) reinforces the importance of these subtype-specific alterations by demonstrating that they are the earliest events in a given IntClust. Notably, however, the gain of 1q happens earlier in IntClust3 tumours (*n* = 80) than IntClust8 (*n* = 57), despite being a common event in both subtypes. It is important to consider that losses cannot be timed using this methodology, and so there may be early subtype-specific losses which we have not detected.

This analysis also revealed additional candidate CNAs of interest in IntClust9 (*n* = 36) and IntClust10 (*n* = 94). In IntClust10 (which comprises mostly triple negative breast cancers), on 8q, we detected early amplification of *MYC*. In IntClust9 (predominantly ER+ tumours but also including a significant number of ER-), on 16q, we observed early gains of *ABCC11*, a member of the Multidrug Resistance Protein family whose expression has previously been associated with an aggressive phenotype and shorter disease-free survival^15^. This is concordant with the high probability of relapse observed in this subtype^5^.

Importantly, this analysis supports several of the IntClust genomic subtypes as being distinct classes that are not fully captured by the PAM50 expression-based subtypes. Replicating our previous findings^6,7^, we found that IntClust classification captures heterogeneity not present in the PAM50 classification (Supplementary Fig. 3). This heterogeneity has previously been shown to be prognostically meaningful^5^. In particular, we here show that IntClust enhances the ability to detect patterns of early gains in 17q23, 11q13-24, 8p12, and 8q—which define IntClust1, IntClust2, IntClust6 and IntClust9 respectively—compared with PAM50 subtype classification (Supplementary Fig. 2b). It is particularly important that these subtypes are treated as separate from other Luminal A/B tumours due to their particularly high risk of relapse for ER+ disease^5^.

### Subtypes are unevenly distributed amongst metastatic sites

**Fig. 1:**
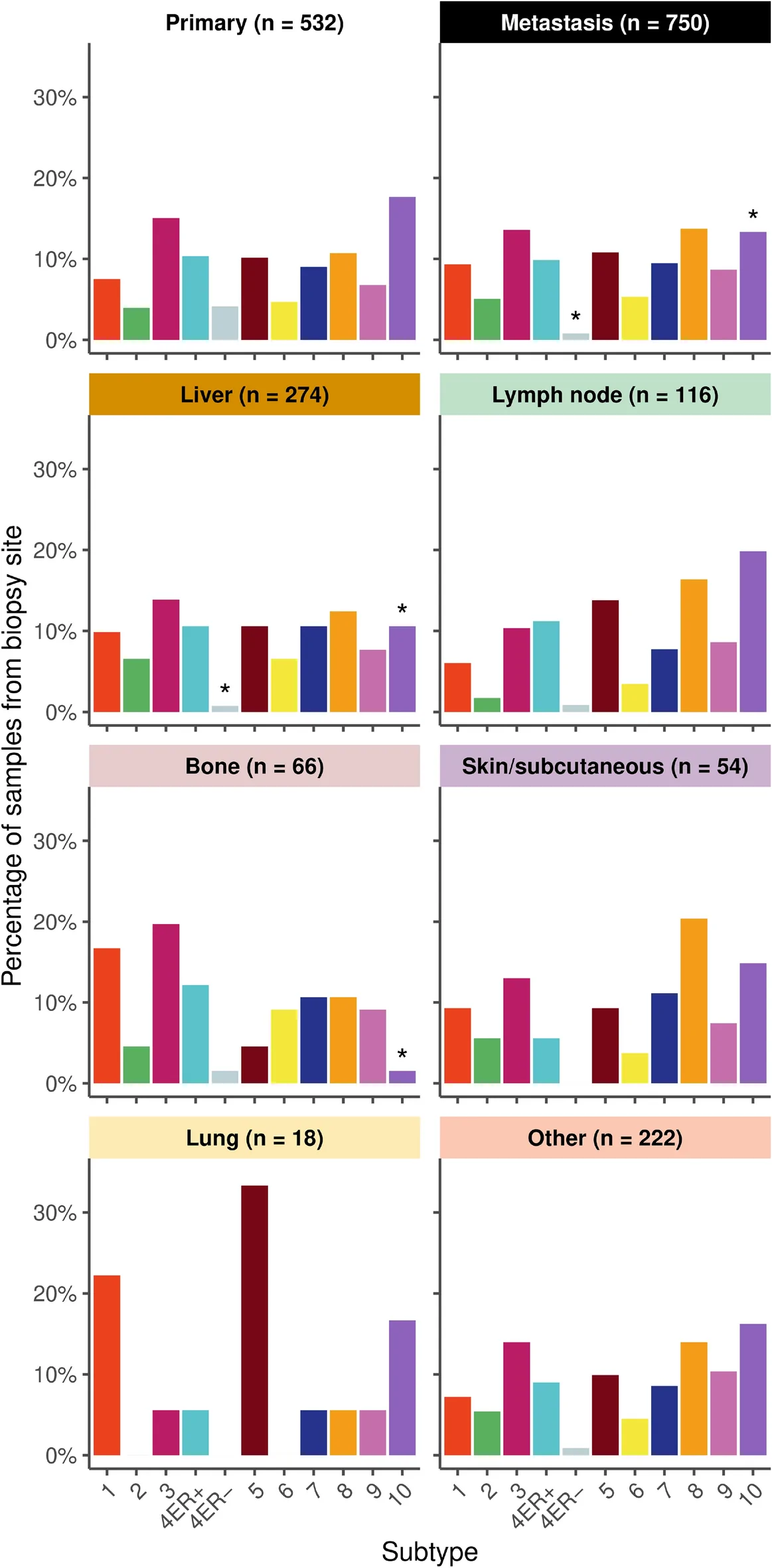
The distribution of breast cancer samples belonging to each genomic subtype in primary breast tumours, breast cancer metastases, and metastases from individual metastatic sites. Asterisks denote where the frequency of a subtype in a metastatic site is significantly different to its frequency in primary breast tumours (binomial test, FDR-adjusted *p* < 0.05).

We next calculated the frequency of breast cancer metastases belonging to each IntClust subtype in each metastatic site, and compared this to each subtype’s frequency in primary breast tumours (Fig. 1). We found a three-fold increase in the proportion of breast cancer metastases belonging to IntClust5 (HER2+) in lung relative to primary breast tumours (6/18; 33.3% *vs.* 54/532; 10.2%); however, this difference was not significant due to the small number of lung metastases in this dataset. The clearest site-specific differences were depletions rather than enrichments: IntClust4ER- tumours were under-represented in liver metastases (2/274; 0.7% *vs.* 22/532; 4.1%), while IntClust10 tumours were under-represented in liver (29/274; 10.6% *vs.* 94/532; 17.7%) and bone (1/66; 1.5% *vs.* 94/532; 17.7%) metastases. Supplementary Fig. 4 shows the same analysis for the PAM50 subtypes; overall, the Her2 and Luminal B subtypes show enrichment in metastases, while the Basal and Luminal A subtypes are depleted.

### Metastases harbour a more homogeneous but unstable genome

**Fig. 2:**
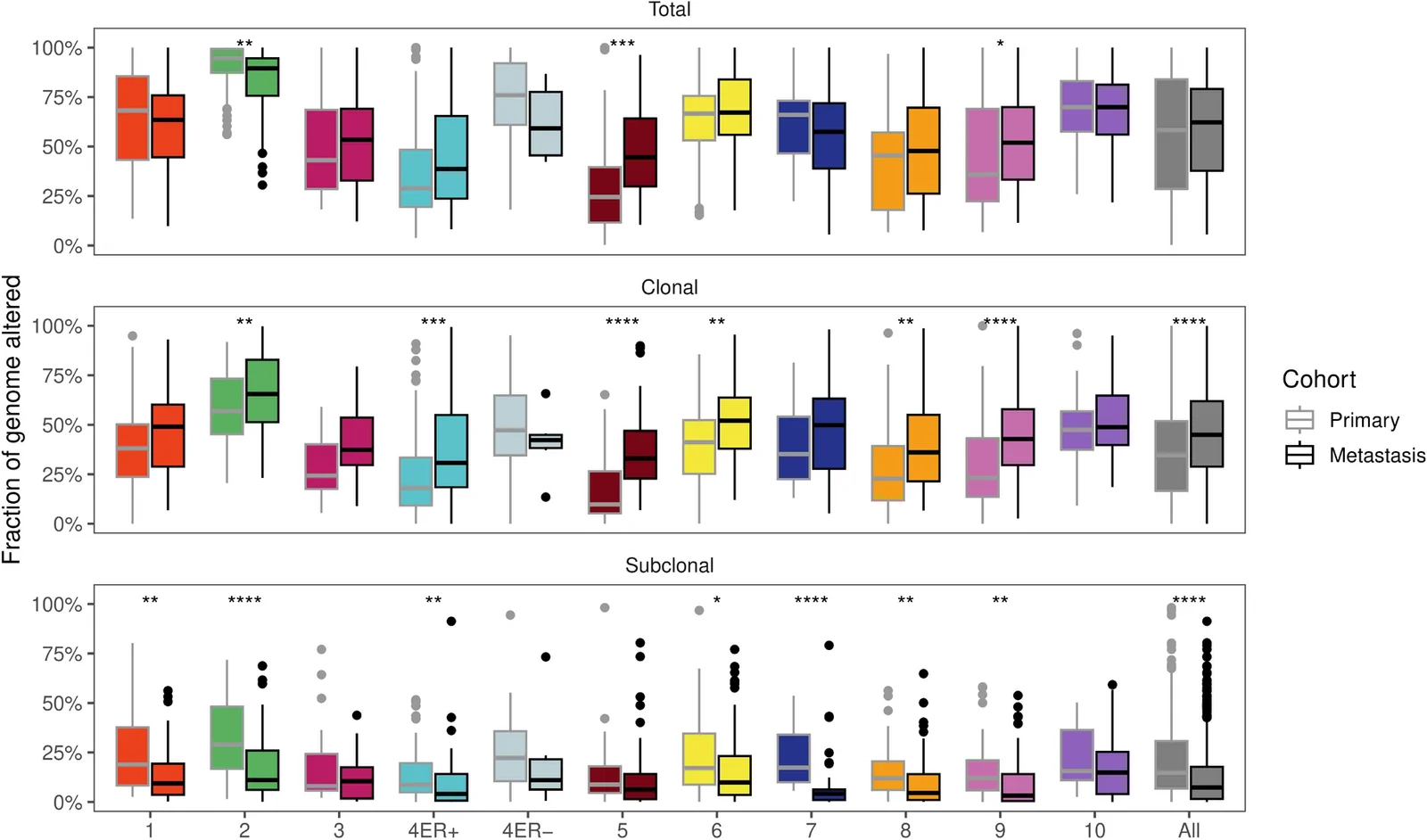
The fraction of genome altered (FGA) by total, clonal and subclonal CNAs across genomic subtypes. FGA was calculated using Battenberg copy number profiles for clonality information. Asterisks denote significant differences between primary tumours and metastases according to Mann–Whitney U tests (FDR-adjusted; *: *p* < 0.05, **: *p* < 0.01, ***: *p* < 0.001, ****: *p* < 0.0001).

When we compared the fraction of genome altered (FGA) by CNAs between primaries and metastases of each subtype (Fig. 2), we observed mixed results, with some subtypes having higher median FGA in metastases than primaries, and *vice versa*. IntClust5 (*n* = 54 primaries, 81 metastases) and IntClust9 (*n* = 36 primaries, 65 metastases) both had significantly higher FGA in metastases, while IntClust2 metastases (*n* = 38) had significantly lower FGA than primaries (*n* = 21).

We then separated CNAs by their clonality^16^ and calculated the FGA by clonal and subclonal CNAs separately. Across all but one of the genomic subtypes, the median clonal FGA was higher in metastases than primaries of the same subtype; this difference reached statistical significance in 7 subtypes. However, the median subclonal FGA was lower in metastases than primaries. Together, the most consistent signal across subtypes was a shift from subclonal to clonal CNA burden in metastases, indicating that metastatic samples are generally more copy-number homogeneous than primary tumours despite retaining substantial genomic alteration. This is in line with previously published pan-cancer studies^12^.

### Subtype-specific enrichment of clonal CNAs between primary tumours and metastases

At the whole-chromosome-arm level, significant differences between primary tumours and metastases of the same subtype were relatively rare (Supplementary Fig. 5a); in IntClust3, gain of 16p was more common in metastases (*n* = 102) than primaries (*n* = 80); similarly, loss of 16p was more common in primaries than metastases. Losses of 17p, 18p and 18q were all more common in IntClust3 metastases than primaries. In IntClust5 tumours, 22p loss was more common in metastases (*n* = 81) than primaries (*n* = 54). All other subtypes showed no significant difference in arm-level CNA frequency.

When comparing aneuploidies in primaries and metastases according to their PAM50 subtype (Supplementary Fig. 5b), we again observed significantly more losses of 18p and 18q in metastases in Luminal A tumours (*n* = 105 primaries, 89 metastases), along with more losses in 1p, 9p, and 11p, and gains in 10p and 13p. We did not observe any significant difference in 22p loss in the Her2 subtype (*n* = 11 primaries, 91 metastases).

However, when we compared primary tumours and metastases while disregarding their subtype, we found many more significant differences in the frequency of arm-level aneuploidy. The most extreme differences were seen in the frequency of 1p, 13q, 22p and 22q losses, and 13p, 16p and 20q gains; all of these events were enriched in metastases (*n* = 750) compared to primaries (*n* = 532).

**Fig. 3:**
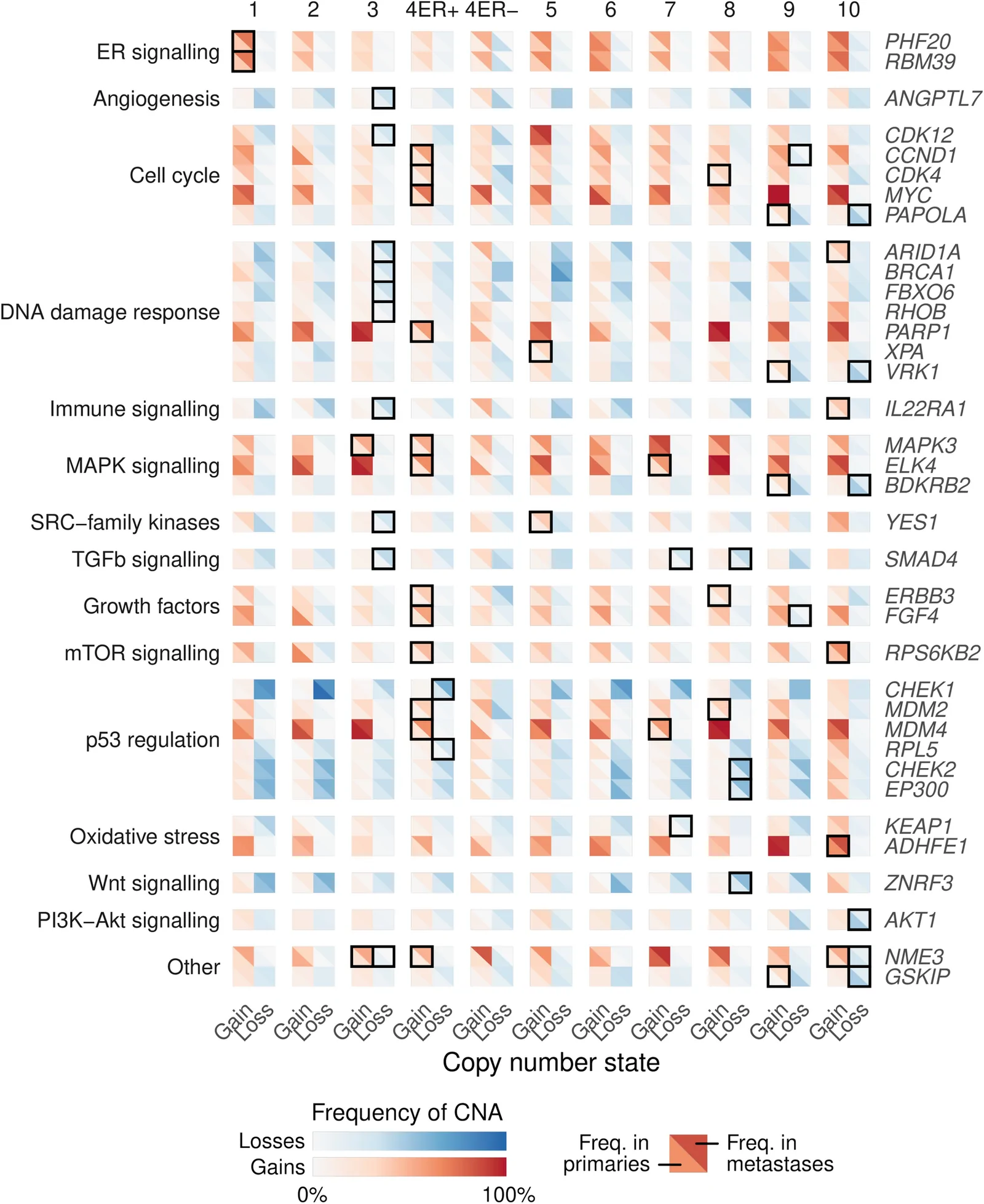
The frequency of CNAs in selected genes and pathways in primary tumours and metastases across genomic subtypes. In each square, the lower and upper triangles’ colours represent the frequency in primary tumours and metastases respectively. Black outlines highlight genes which fall into genomic regions which have significantly different alteration frequencies between primary tumours and metastases according to a Fisher test (FDR-adjusted, *p* < 0.05).

Next, we compared the frequency of CNAs in primary tumours and metastases (Supplementary Fig. 6) in bins across the genome in order to identify regions with differential copy number. Supplementary Fig. 7 shows the adjusted *p*-value of Fisher’s exact tests applied to each bin, while Fig. 3 shows a selection of genes falling in genomic regions where the difference in frequency was significant. The strongest subtype-specific signals implicated different biological processes in different genomic backgrounds, including ER signalling in IntClust1, DNA repair and HRD-related genes in IntClust3, MAPK and p53-associated alterations in IntClust4ER+ and IntClust8, and therapy-resistance or stress-response candidates in IntClust5, IntClust9 and IntClust10.

In IntClust1 (*n* = 40 primaries, 70 metastases), gains of two ER*α* co-activator genes, *RBM39* (CAPER)^17^ and *PHF20*^18^, were observed significantly more often in metastases than primaries. This raises the possibility that increased ER*α* activation may contribute to endocrine therapy resistance in IntClust1 metastases.

In IntClust3 tumours (*n* = 80 primaries, 102 metastases), several tumour suppressor genes were significantly more likely to be lost in metastases than primaries. These included *ARID1A*, *BRCA1*, *FBXO6* and *RHOB*. Given the key roles these genes play in homologous recombination (HR) repair of double-stranded breaks^19–21^ and the DNA damage response, this is consistent with the hypothesis that HR defects may be associated with metastatic progression in IntClust3 tumours. Previous work has shown that brain metastases from breast cancer show increased HR deficiency (HRD) scores compared to their matched primaries^22^. When we calculated HRD scores in the two cohorts, we observed significantly higher scores in IntClust3 metastases than primaries (Supplementary Fig. 8). This was also the case for IntClust4ER+, IntClust5, IntClust7 and IntClust8. Interestingly, the predominantly triple-negative IntClust10 subtype had significantly lower HRD scores in metastases than primaries.

In addition, *ANGPTL7*—an anti-angiogenic gene whose upregulation has been shown to decrease hepatic metastasis formation in a mouse model^23^—was shown to be significantly more commonly lost in IntClust3 metastases than primaries. This may represent one candidate contributor to the high frequency of IntClust3 metastases found in the liver (Fig. 1).

The oncogenic growth factor *FGF4* and receptor *ERBB3* were significantly more likely to be gained in IntClust4ER+ metastases (*n* = 74) versus primaries (*n* = 55), along with downstream effectors in the MAPK pathway *MAPK3* (*ERK1*) and *ELK4*. Three targets of the MAPK pathway, key cell cycle regulators *CCND1*, *CDK4* and *MYC*, were also more commonly gained in metastases. This was paired with significantly more frequent loss of *CHEK1* and *RPL5* and gains of *MDM2* and *MDM4*, indicating possible downregulation of p53 cell cycle arrest activity^24–27^.

In the predominantly HER2+ IntClust5 group (*n* = 54 primaries, 81 metastases), significantly more gains of *YES1* and *XPA* were observed in metastases than primaries. In several tumour types, *YES1* has been linked to trastuzumab resistance^28^, while higher *XPA* expression is associated with decreased cisplatin sensitivity^29–32^.

When comparing IntClust7 primaries (*n* = 48) and metastases (*n* = 71), significantly more losses of *KEAP1* and gains of *MDM4* were observed in metastases, which may allow tumours to tolerate oxidative stress^33^ and manage the resulting p53 activity that would normally lead to cell cycle arrest or apoptosis^27^.

In IntClust8 metastases (*n* = 103 metastases, 57 primaries), more frequent losses of p53 co-activator *EP300*^34^, losses of *CHEK2*^35^ and gains of *MDM2*^26^ may be associated with decreased p53 activity. This is alongside more frequent gains in *CDK4* and *ERBB3*, indicating increased growth signalling and subsequent cell cycle progression.

In IntClust9 (*n* = 36 primaries, 65 metastases), the CNAs enriched in metastases impact multiple pathways. Gains in *VRK1* are a candidate mechanism that could increase metastatic potential, as previously observed^36^, as it confers resistance to DNA damaging agents in breast cancer^37^ thanks to its role in DNA damage repair^38^. Gains were also observed in the bradykinin receptor *BDKRB2*, which may increase MAPK signalling^39^. Proliferation could also be stimulated more directly by gains of *PAPOLA*, which encodes a protein responsible for the addition of poly(A) tails to mRNA transcripts, and which promotes cyclin D upregulation. Gains were additionally seen in *GSKIP*, a scaffold protein for a multifunctional serine/threonine kinase GSK3*β*. GSK3*β* expression is associated with poor prognosis in early breast cancer patients^40^, but its role as a tumour suppressor or oncogene is debated and appears to be context-specific^41^.

Several of the same genes that were more frequently gained in IntClust9 metastases than primaries were instead more frequently lost in primaries (*n* = 94) than metastases (*n* = 100) when looking at IntClust10 tumours (*PAPOLA*, *VRK1*, *BDKRB2* and *GSKIP*). Additionally, in IntClust10, significantly more frequent gains of *RPS6KB2*, a downstream effector of mTOR, were observed in metastases. Its gain is predictive of poor prognosis in breast cancer^42^. Gains in the ROS-promoting mitochondrial enzyme *ADHFE1*^43^ were also observed. Loss of *NME3* and the oncogene *AKT1* was also observed less commonly in IntClust10 metastases than primaries; while *NME3* itself has not been shown to be a tumour suppressor, several members of the NME family have^44^.

When making the same comparison between primary tumours and metastases according to their PAM50 subtype, we observe many of the same regions had differential copy number as with the IntClust genomic subtypes (Supplementary Fig. 7b). Differences specific to genomic subtypes, however, coalesced into the same PAM50 subtype; for example, losses in distal 1p and 8 (IntClust3), proximal 12p gains (IntClust8), and several alterations in IntClust4ER+ were all observed as being differentially altered in the Luminal A subtype (*n* = 105 primaries, 89 metastases).

### Arm-level alterations reveal additional candidate loci associated with selection in metastases

While our analyses of arm-level events revealed a handful of subtype-specific differences that may be associated with metastatic potential, determining which loci on the arm may contribute to any fitness advantage can be challenging. We addressed this problem by analysing events dubbed partial-sCNAs—somatic copy number alterations that are bound by either the telomere or centromere—using the tool BISCUT^45^. BISCUT uses the length distributions of partial-sCNAs to identify genomic loci under positive or negative selection relative to a reference dataset. In the original analysis by Shih *et al.*^45^, this reference dataset comprised all chromosome arms and cancer types in TCGA. Because we are interested in loci that may be associated with metastatic fitness within a given subtype, we generated reference length distributions that were specific to each arm in each subtype using primary tumours from ICGC and METABRIC^6^, which was added to achieve an acceptable sample size for generating breakpoint distributions.

**Fig. 4:**
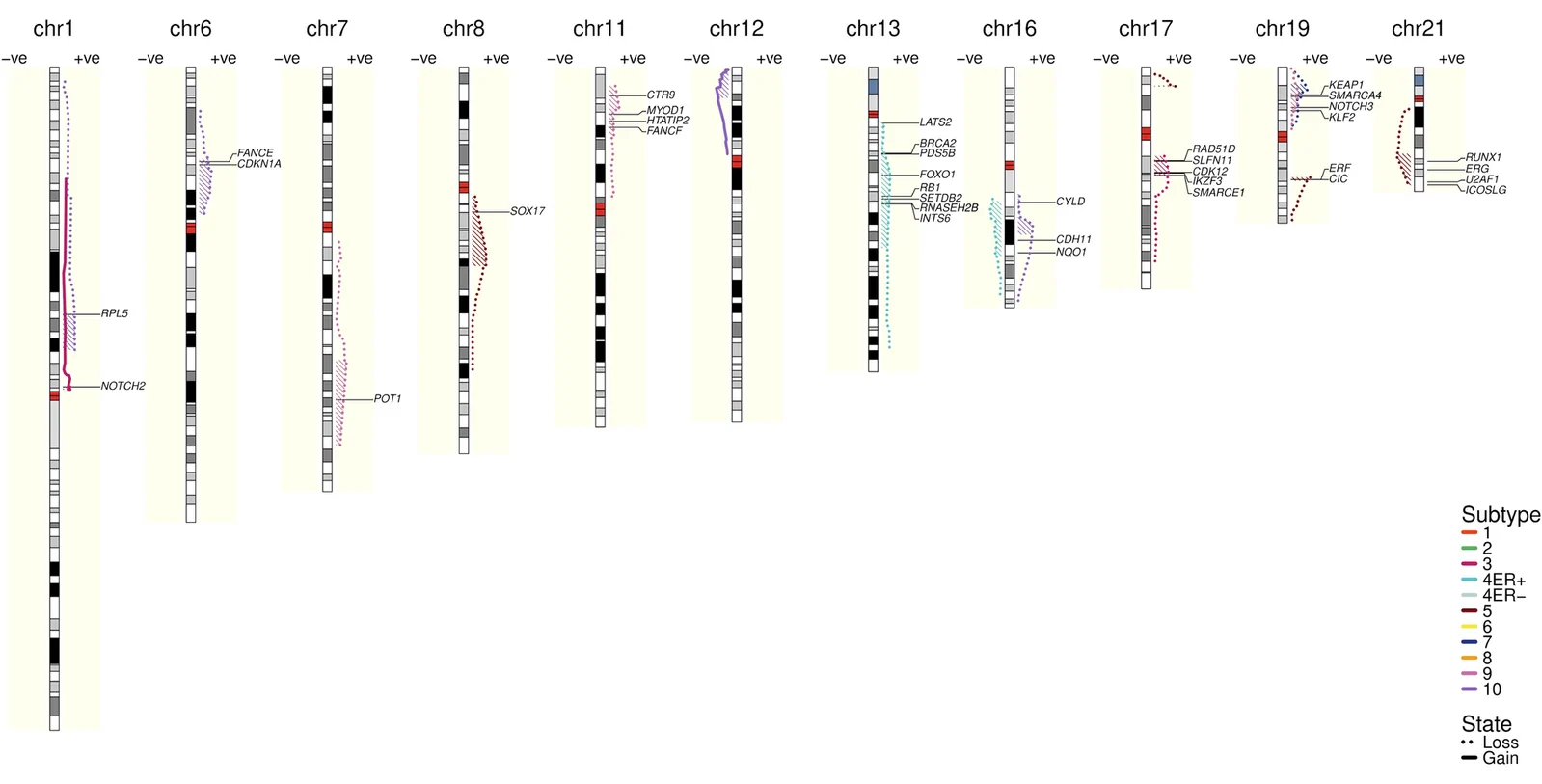
Selection peaks detected with BISCUT breakpoint analysis. Lines’ distance from each chromosome represents the distance between the cumulative breakpoint distributions of primary tumours and metastases. Lines to the left and right of chromosomes represent negative and positive selection, respectively, in metastases; solid and dashed lines represent selection of gains and losses respectively; and colours represent subtype. Hatched areas indicate the location of significant peaks as calculated by BISCUT. The locations of genes in the OncoKB database which fall within significant peaks are indicated.

This analysis revealed additional loci of interest not identified by the focal CNA analysis (Fig. 4). These peaks expanded the candidate landscape beyond focal CNAs, highlighting broader partial-arm events affecting DNA repair loci in IntClust3 and IntClust4ER+, ETS/MAPK-related loci in IntClust5, and stress-response genes in IntClust10.

In ER+ IntClust4 metastases (*n* = 74), we observed “tumour suppressor-like selection”—*i.e.* positive selection of deletions—across a long region of 13q. Genes in this region include several important tumour suppressors involved in the repair of DNA damage: *BRCA2*, and a BRCA2-interacting protein *PDS5B*^46^; *RNASEH2B*, whose loss impairs nucleotide excision repair and increases PARP trapping, leading to increased genomic instability^47^; relatedly, *RB1*, loss of which restores PARP inhibitor resistance in cells made sensitive by *RNASEH2B* loss^47^; and *FOXO1*, which is important in promoting DNA damage-induced cell death^48^.

IntClust3 metastases (*n* = 102) also showed tumour suppressor-like selection for genes involved in DNA repair and replication stress: homologous recombination repair protein *RAD51D*^49^; *CDK12*, which regulates the expression of several DNA damage response genes^50^; *SMARCE1*, a subunit of the SWI/SNF chromatin remodelling complex^51^; and *SLFN11*, which triggers cell death in response to stressed replication forks and inhibits homologous recombination repair.

In the predominantly HER2+ IntClust5 subtype (*n* = 81), members of the ETS family and their regulators were under selection. *ERF*— which controls cell proliferation at the G0/G1 transition and is a target of MAPK signalling^52^—and *CIC*, which regulates several members of the ETS family^53^ and is a negative regulator of MAPK signalling^51^, showed tumour suppressor-like selection. *ERG*, another ETS family member, is an oncogene that increases cell invasion^54^, and was subject to essential-like selection (negative selection of losses). Interestingly, loss of *ICOSLG* was also negatively selected for in IntClust5 metastases: high expression of *ICOSLG* has been identified as a potential biomarker of trastuzumab resistance^54^.

In IntClust10 (*n* = 100), two genes that were subject to tumour suppressor-like selection play key roles in cellular responses to stress: *RPL5* activates p53 activity when triggered by ribosomal stress^25^, and while *CYLD*’s most-characterised role in cancer is as a negative regulator of the NF*κ*B pathway^55^, it may also promote oxidative stress^56^.

When we repeated this analysis using the PAM50 subtypes, we found that the identified peaks were broadly quite different from those found using the genomic subtypes (Supplementary Fig. 9), though there was some overlap; namely, positive selection for losses in the Her2 and IntClust5 subtypes in 19q, losses in the Basal and IntClust9 subtypes on 11p, and gains in the Luminal A and IntClust3 subtypes on 1p. The difference in selection peaks between the PAM50 expression subtypes and the genomic subtypes may indicate that both the transcriptomic and genomic background of a primary tumour are important factors in determining which partial-sCNAs increase fitness to metastasise.

## Discussion

Overall, our analyses support an association between the background genomic landscape of a primary tumour and the enrichment or depletion of specific CNAs in metastases. These CNAs affect pathways that may contribute to metastatic potential.

Our re-processing of raw sequencing data using a unified pipeline was designed to reduce biases introduced by different choices of computational methods between the primary (ICGC) and metastatic (HMF) studies. However, this harmonisation cannot eliminate all residual cohort and technical confounding. Because our analysis used primary tumours and metastases from separate cohorts to achieve greater sample sizes than have been possible with paired samples, differential copy number may reflect differences in study populations, sequencing protocols, treatment-induced clonal selection, site-specific evolution, sampling bias, or metastatic biology itself.

However, promisingly, we do find support for several of our findings in previous studies of paired samples: *CCND1* and *CDK4* gains were observed in brain metastases by Brastianos *et al.*^57^; enrichment in metastases of *MYC* gains in triple-negative cases, and *CCND1* and *MDM4* gains in ER+ cases was reported by Aftimos *et al.*^58^; lastly, Poplawski *et al.*^59^ identified a case of concurrent 8p12/11q13 gain in metastasis—two CNAs which were enriched in our metastases cohort—and a case of concurrent 1p/16p gain, characteristic of the IntClust4ER+ metastases in the present study.

The metastatic dataset is also inherently biased by differences in the feasibility of obtaining biopsies from different metastatic sites in patients. In addition, most patients only had one metastatic lesion biopsied, even if tumours were detected in multiple sites. Our previous work examining metastases obtained at autopsy^10^, where samples could be obtained from all metastatic sites, revealed that known genomic driver events are overwhelmingly shared between metastases across different sites, and so are likely to be the result of a single seeding event. This suggests that the same metastasis-associated CNAs identified by our analyses are likely also present in under-represented metastasis sites.

While PAM50 subtypes capture transcriptional programmes with established clinical relevance, IntClust captures genomic architectures that further stratify breast cancers within clinical and PAM50 groups. The clinical relevance of this genomic stratification has been supported by validation across large primary breast cancer cohorts, including the original expanded IntClust classifier analysis^7^, long-term relapse modelling showing subtype-specific risks of late recurrence^5^, recent WGS analyses linking genomic architecture to evolutionary trajectories and treatment-relevant features^60,61^, and neoadjuvant response modelling in which IntClust contributed to therapy-response prediction^62^.

The use of WGS in this study, as opposed to targeted sequencing, has the distinct benefit of allowing us to assess genes without previous strong evidence of association with metastasis. While we have attempted to discern the functional relevance of genes in differential copy number regions using existing literature, it is quite likely that other genes we have not named here are relevant to the metastasis-associated CNA patterns we report. External validation of these subtype-specific CNA patterns is necessary, but suitable large WGS cohorts of breast cancer metastases are not yet available. The candidate mechanisms nominated here therefore require functional validation beyond the scope of this study before their biological or therapeutic relevance can be established.

Based on the strength of the subtype-specific signals, prior biological evidence, and potential clinical relevance, we consider several hypotheses to be particularly high priorities for functional validation: whether DNA repair gene losses in IntClust3, and related partial-arm events in IntClust4ER+, generate therapeutically exploitable HRD; whether *ERBB3* gains in IntClust4ER+ and IntClust8 create dependency on HER3/MAPK signalling; whether *YES1* gains in IntClust5 contribute to resistance to HER2-targeted therapy; and whether *RPS6KB2* and *ADHFE1* gains in IntClust10 create targetable mTOR or metabolic-stress dependencies.

## Methods

### Sample cohorts

Requests for access to WGS and RNA-seq alignment files from breast cancer patients were made to the International Cancer Genome Consortium Data Access Compliance Office (application references *DACO-259* and *DACO-2103*, BRCA-EU project,^63^) and the Hartwig Medical Foundation (HMF) Data Access Board (application references *DR-129*, *DR-175* and *DR-287*, all breast cancer patients,^64^). For the ICGC BRCA-EU dataset, alignment files were downloaded for 551 primary breast tumour samples with 551 associated normals from 551 patients from the European Genome-Phenome Archive (EGA). For the HMF dataset, alignment files were downloaded for 791 breast cancer metastasis samples with 758 associated normals from 758 patients from the Google Cloud Platform. All comparisons between ICGC and HMF samples were performed after reprocessing with the same alignment and CNA calling pipeline described below, to minimise technical differences.

### WGS re-alignment to GRCh38

Re-alignment of the GRCh37 alignment files to GRCh38 was performed according to GATK Best Practices for Data Pre-Processing for Variant Discovery. The reads were extracted from the input alignment files using GATK SamToFastq. These reads were then piped into BWA-MEM 0.7.15-r1140 to align them to GRCh38 (excluding chrY, as all patients were female). Output SAM alignments were piped into SAMtools to convert them to BAM files. The BAM files were sorted with SAMtools and duplicates were marked with GATK MarkDuplicates. Base quality score recalibration was performed using GATK BaseRecalibrator and ApplyBQSR as this has been shown to improve downstream variant calling.

### RNA-seq processing for PAM50 classification

RNA-seq data for 226 primary tumours and 384 metastases were aligned using STAR v2.7.11^65^, and gene-based transcript abundance was estimated using Salmon v1.4.0^66^ in alignment-based mode. We normalised primary and metastatic samples using three post-processing steps. First, we employed the edgeR R package^67^ to estimate effective library sizes that account for sequencing depth and RNA composition. Second, to scale the normalised count data for linear modelling, the counts were transformed into log2 counts per million and assigned proper weights using the voom method in the limma R package^68^. Third, technical effects related to differences in studies were corrected using ComBat from the sva R package^69^. Finally, library and gene lengths were also corrected for in the normalisation process. PAM50 classification was performed using the genefu R package^70^.

### Small variant calling

Somatic and germline small variants (SNVs, MNVs and indels) are used as an input to Canvas copy number calling, while Purple requires somatic variants. Hence, Mutect2 from GATK (v4.2.6.1) was used for small variant calling. First, indels from Mutect2 were realigned so that they were consistent for each region (BWA aligns reads independently of each other, so each read can align differently around the same insertion or deletion).

Then, in order to filter possible sequencing artefacts, we created a panel of normals (PoN) for each sample cohort. This procedure uses all of the variant calls from normal samples to create a panel of variants that are likely to be recurrent technical artefacts. Mutect2 was run in artefact detection mode on the whole set of normal BAM files for each cohort, and the results passed to GATK CreateSomaticPanelOfNormals, with variants being required to appear in at least 2 samples to be considered for the panel.

Somatic variants were finally called by Mutect2, and variants in the PoN were filtered from the final set of somatic mutations. Germline variants were called with GATK HaplotypeCaller.

### Copy number consensus

Our approach to generating a copy number consensus was inspired by the work previously performed by the PCAWG consortium^71^.

Briefly, this approach comprises applying the following steps to each tumour sample individually:

1. Running multiple copy number callers
2. Generating a consensus of segment coordinates/breakpoints
3. Mapping the copy number states from (1) to the consensus segments from (2)
4. Generating a consensus copy number state for each consensus segment using a voting strategy

Each of these steps is described in more detail below.

### Running multiple copy number callers

We ran three copy number callers on all samples: Canvas (Somatic-WGS)^72^, Purple^64^ and Battenberg^16^. We overrode default settings to specify a maximum absolute ploidy of 5 for all callers in order to minimise variation arising from the different default values of this parameter.

### Generating a consensus of segments/breakpoints

The output of each caller comprises a list of major and minor copy number states, each spanning a contiguous range of genomic coordinates, *i.e.* a segment. Each end of this segment is a breakpoint where the copy number state changes. Each caller outputs different breakpoints. Hence, to calculate a consensus copy number profile for each sample, we firstly had to generate a set of consensus breakpoints for each sample.

To achieve this, we adapted a previously published consensus segmentation algorithm^73^, and applied it to each tumour sample. As this algorithm was designed for transcriptomic data, we found it was prohibitively computationally inefficient for calculating consensus segments for WGS data. Hence, to increase the efficiency of the algorithm, we replaced the genomic distance between breakpoints with the rank of that distance.

### Mapping copy number states to consensus segments

Subsequently, we truncated any consensus segments which overlapped telomeres or centromeres, where copy number calling is less reliable. We then re-mapped copy number states from each caller to each consensus segment by choosing the original caller’s segment that had the highest overlap (in terms of genomic coordinates) with each consensus segment, and assigning that original segment’s copy number state to the consensus segment. This resulted in each consensus segment having a set of three possible copy number states (one from each caller).

### Generating a consensus copy number state for each consensus segment

The final step required the assignment of a consensus copy number state to each consensus segment. We counted the number of callers which agreed (“voted”) on the overall (major and minor) integer copy number state for each consensus segment, and the state with the highest number of votes was assigned to that consensus segment. In the rare cases where one or more callers did not output a state for a particular consensus segment, we assigned the state from the caller with highest agreement with the consensus states where all callers’ votes were available, in line with Dentro *et al.*^71^.

### IntClust classification

We utilised the CopyClust classifier^14^ to classify the samples into IntClust subtypes using their consensus copy number profiles. As the classifier requires copy number log ratios as input, we calculated these from consensus copy number profiles using the following approach.

### Consensus purity & ploidy

We calculated each tumour’s ploidy *Ψ* as the mean consensus copy number weighted by segment length. We then calculated a purity estimate by taking the mode of the distribution of *ρ* over all segments *s*, each having consensus copy number $T_{s}$ and copy number log ratio $r_{s}$ (as calculated by Battenberg^16^):

$$\begin{aligned} \rho = \text {mode}\left( \frac{2-2r_{s}}{\Psi r_{s} - 2r_{s} + 2 - T_{s}} \right) \end{aligned}$$

### Back-calculate consensus copy number log ratios

Subsequently, we calculated consensus log ratios *R* for each segment *s* to input into the CopyClust classifier as:

$$\begin{aligned} R_{s} = log_2\left( \frac{2(1-\rho )+T_{s}\rho }{2(1-\rho )+\Psi \rho }\right) \end{aligned}$$

Finally, we quantile normalised each feature’s log ratios within each cohort, using the log ratios from METABRIC as reference distributions.

### Site/subtype post-hoc power analysis

Using the site/subtype comparisons shown in Fig. 1, we calculated post-hoc power for the same two-sided exact binomial test used to compare subtype frequencies between primary tumours and metastases. The primary cohort subtype fraction was used as the null proportion, the observed metastatic-site subtype fraction was used as the alternative proportion (effect size), and the metastatic-site sample size was used as *n*. For each comparison, we enumerated all possible metastatic subtype counts from 0 to *n* and identified the counts that would reject the null hypothesis in an exact binomial test. Power was then calculated as the probability, under a binomial distribution parameterised by the observed metastatic-site subtype fraction, of observing a count in this rejection region. To account for multiple testing, the significance threshold used to define the rejection region was Bonferroni-corrected as *0.05 / m*, where *m* was the number of site/subtype comparisons. Comparisons with corrected post-hoc power >= 0.8 were classified as powered to detect the observed effect size; those were:

**Table:** 

Site	Subtype
All metastases	4ER-
All metastases	LumB
All metastases	Basal
All metastases	Her2
All metastases	LumA
Liver	Her2
Liver	LumA
Liver	LumB
Liver	Basal
Lymph node	Her2
Lymph node	LumA
Bone	10
Skin/subcutaneous	Her2
Other	Her2

### Primary vs. metastasis bin-level comparison

To identify regions of differential copy number between primary tumours and metastases, we generated bins by taking the union of consensus breakpoints for all samples. For each bin, we then calculated the number of primary and metastatic samples with gains and losses in each bin. Gains and losses were counted when integer copy number of a segment was greater or less than the integer ploidy of the sample, respectively. We then performed Fisher tests for each bin, to calculate a *p*-value for the difference in the frequency of gains and losses between primaries and metastases. Finally, we performed FDR adjustment of the resulting p-values to correct for multiple testing.

### Primary vs. metastasis aneuploidy comparison

Aneuploidy was called at arm level based on greater than 80% of an arm’s length having a copy number state different from the rounded consensus ploidy.

### Copy number timing

To generate estimated mutation times for CNAs, we used the MutationTimeR software^74^. This software requires input from the Battenberg subclonal copy number caller, and the subclonal decomposition of SNVs. We generated this second input using DPClust3p^16^ to generate cancer cell fractions for SNVs, followed by Mobster^75^ to remove neutral tail SNVs, and finally DPClust^16^ to generate the subclonal decomposition of SNVs.

The output of MutationTimeR is a mutation time in a range from 0 to 1 (early to late) for each alteration. To calculate a normalised average timing of gains within each subtype in bins across the genome, we used a weighted least squares approach that accounts for alteration frequency (both absolute and relative to subtype frequency) and the overlap of each segment with each bin. This approach shrinks timings of alterations that occur in a low proportion of samples, or in a low absolute number of samples, towards 0.5.

For each subtype *i* and each bin *j*, the normalised average time $T_{ij}$ is given by:

$$\begin{aligned} T_{ij} = (1-\frac{1}{n_{ij}}) \hat{\tau }_{ij} + \frac{1}{2n_{ij}} \end{aligned}$$

where $n_{ij}$ is the number of samples with a timed alteration in that subtype and bin, and

$$\begin{aligned} \hat{\tau }_{ij} = \mathop {\textrm{argmin}}\nolimits _{\tau _{ij}} \sum _{k_{ij}} w_{k_{ij}} \left[ \tau _{ij} - \left\{0.5+\rho _{ij}(t_{k_{ij}}-0.5)\right\} \right] ^2 \end{aligned}$$

where $k_{ij}$ are the segments overlapping the bin in that subtype, $w_{k_{ij}}$ is the length of overlap between each segment and bin, $\rho _{ij}$ is the proportion of samples in that subtype that have a timable alteration in that bin, and $t_{k_{ij}}$ is the mutation time estimate output by MutationTimeR for each segment.

### HRD scores

HRD scores, as defined in Telli *et al*^76^, were obtained by summing the loss of heterozygosity, large-scale transition and telomeric allelic imbalance scores as calculated by the signature.tools.lib R package^77,78^.

### BISCUT analysis

Background distributions of breakpoints were generated separately for each chromosome arm in each subtype using the consensus copy number calls for ICGC samples and the published METABRIC segmentation files. Foreground distributions were generated similarly using Hartwig Medical Foundation (metastasis) samples. The distance between distributions, and the peak locations, were then calculated using BISCUT^45^. Peaks supported by fewer than 10 samples in the foreground or background distributions were removed.

### Analysis code and software dependencies

Containers and Nextflow pipelines have been made available on Zenodo (as detailed in *Code availability*), which contain the dependencies and code used to generate the results and figures from this paper.

## Supplementary Information

Supplementary Information.

## Data Availability

Sequencing data for primary tumours is available subject to authorization by the ICGC Data Access Compliance Office (https://daco.icgc-argo.org/). Sequencing data for metastases is available subject to authorization by Hartwig Medical Foundation (https://www.hartwigmedicalfoundation.nl/en/data/data-access-request/). Molecular data for METABRIC is deposited at the European Genome-Phenome Archive (study EGAS00000000083) and is available upon request to the METABRIC data access committee.

## Code availability

All original code has been deposited at Zenodo (DOI: https://doi.org/10.5281/zenodo.15603433), and is publicly available as of the date of publication. This includes container definition files for recreation of the correctly-versioned software environment, as well as Nextflow pipelines housing all analysis and figure-generation code giving all parameters used to generate the data presented in this manuscript.

## Materials availability

This study did not generate new tumour samples.
