# Supplementary material for: Breast cancer genomic subtype-specific copy number alterations in metastases
Source: Sci Rep. 2026 Jul 11;16:31934. doi: 10.1038/s41598-026-60972-2 (PMC13658008; doi:10.1038/s41598-026-60972-2)
Supplement: Supplementary file 1 [file 41598_2026_60972_MOESM1_ESM.pdf]

Supplementary Tables

Supplementary Table 1: Clinical and molecular characteristics of the primary tumour and metastatic cohorts.

	**ICGC**<br>**Primary tumours**<br>**n = 532**	**Hartwig Medical Foundation**<br>**Metastases**<br>**n = 750**
**Age**	Mean: 55.3 (SD: 13.5)	Mean: 57.2 (SD: 11.0)
**Sex**	Female: 532 (100%)	Female: 694 (92.5%)<br>Missing: 56 (7.5%)
**Stage**	I: 61 (11.5%)<br>II: 150 (28.2%)<br>III: 56 (10.5%)<br>IV: 3 (0.6%)<br>Missing: 262 (49.2%)	Metastatic: 655 (87.3%)<br>Locally advanced: 36 (4.8%)<br>Missing: 59 (7.9%)
**Grade**	I: 39 (7.3%)<br>II: 154 (28.9%)<br>III: 264 (49.6%)<br>Missing: 75 (14.1%)	Missing: 750 (100%)
**IntClust**	1: 40 (7.5%)<br>2: 21 (3.9%)<br>3: 80 (15.0%)<br>4ER-: 22 (4.1%)<br>4ER+: 55 (10.3%)<br>5: 54 (10.2%)<br>6: 25 (4.7%)<br>7: 48 (9.0%)<br>8: 57 (10.7%)<br>9: 36 (6.8%)<br>10: 94 (17.7%)	1: 70 (9.3%)<br>2: 38 (5.1%)<br>3: 102 (13.6%)<br>4ER-: 6 (0.8%)<br>4ER+: 74 (9.9%)<br>5: 81 (10.8%)<br>6: 40 (5.3%)<br>7: 71 (9.5%)<br>8: 103 (13.7%)<br>9: 65 (8.7%)<br>10: 100 (13.3%)
**PAM50***	Basal: 48 (22.3%)<br>Her2: 11 (5.1%)<br>LumA: 105 (48.8%)<br>LumB: 49 (22.8%)<br>Normal: 2 (0.9%)	Basal: 50 (13.6%)<br>Her2: 91 (24.8%)<br>LumA: 89 (24.3%)<br>LumB: 129 (35.1%)<br>Normal: 8 (2.2%)

*: Only for 215 primary and 367 metastatic samples with gene expression data

Supplementary Figures

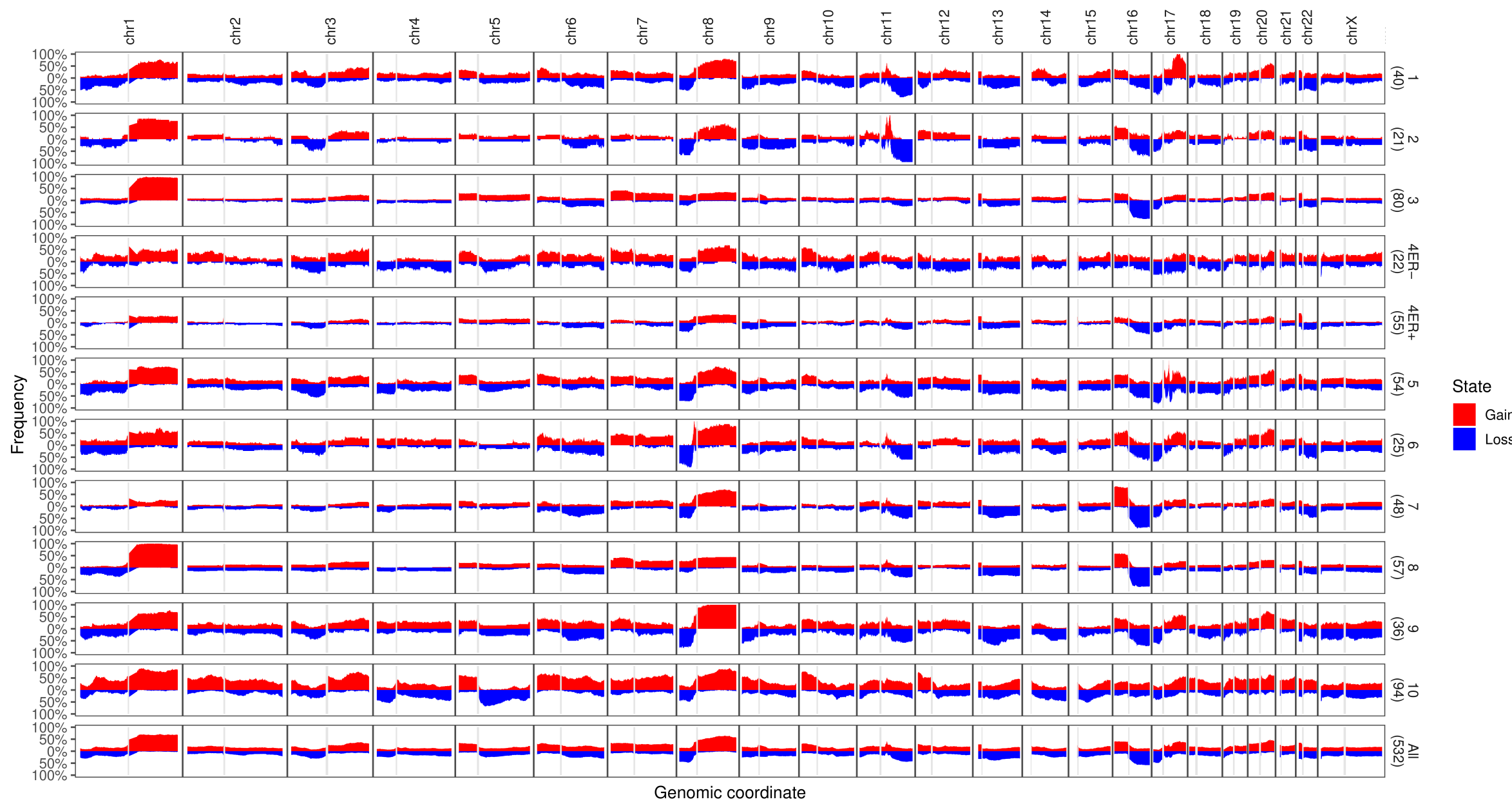

(a) Genomic subtypes

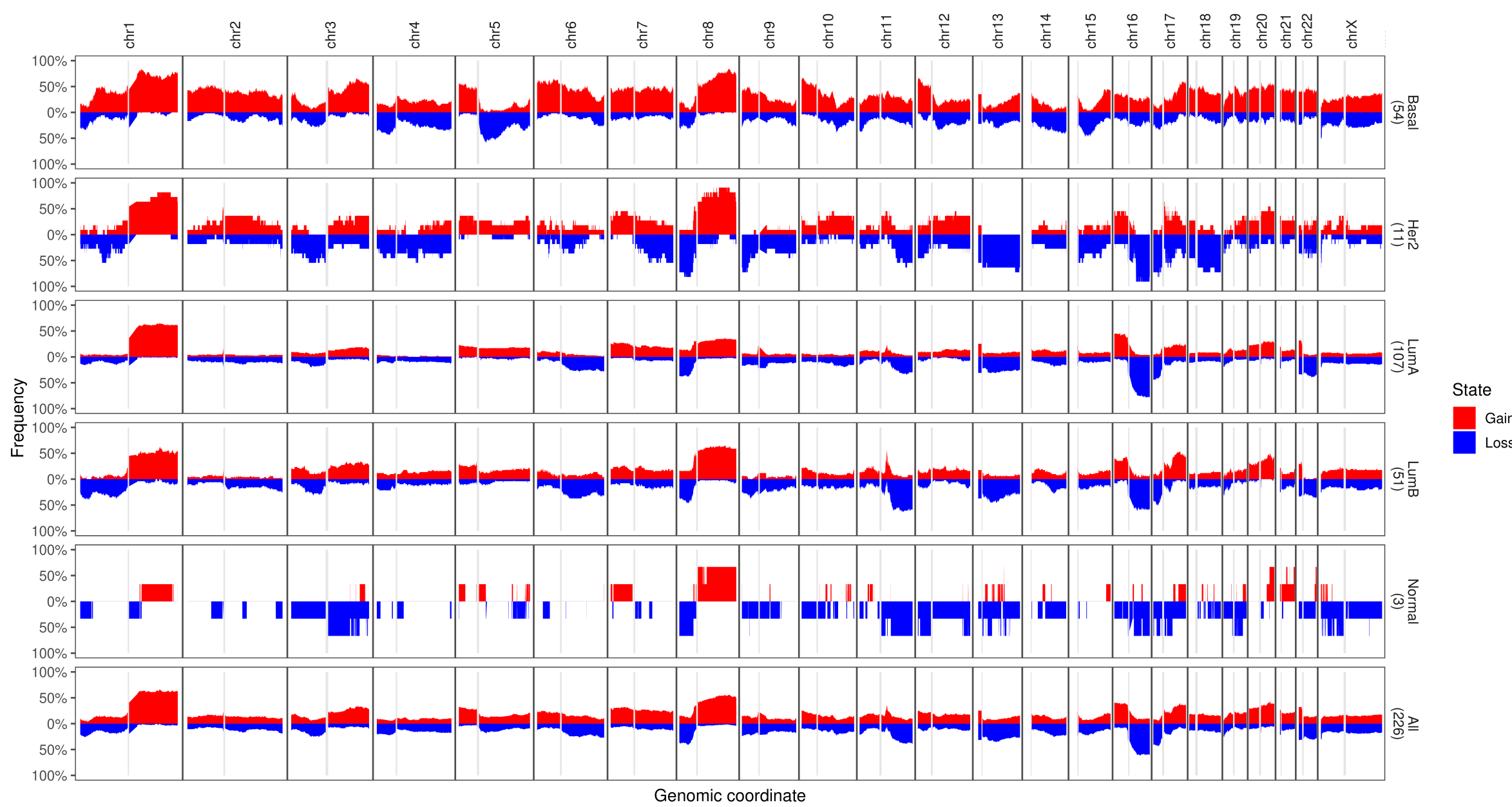

(b) PAM50 subtypes

Supplementary Figure 1: The frequency and genomic distribution of CNAs in primary tumours across subtypes. The frequency of gains is represented by the red shaded area above the zero line; the frequency of losses is represented by the blue shaded area below the zero line. Light grey boxes indicate the location of centromeres. The number of samples in each subtype is indicated in brackets.

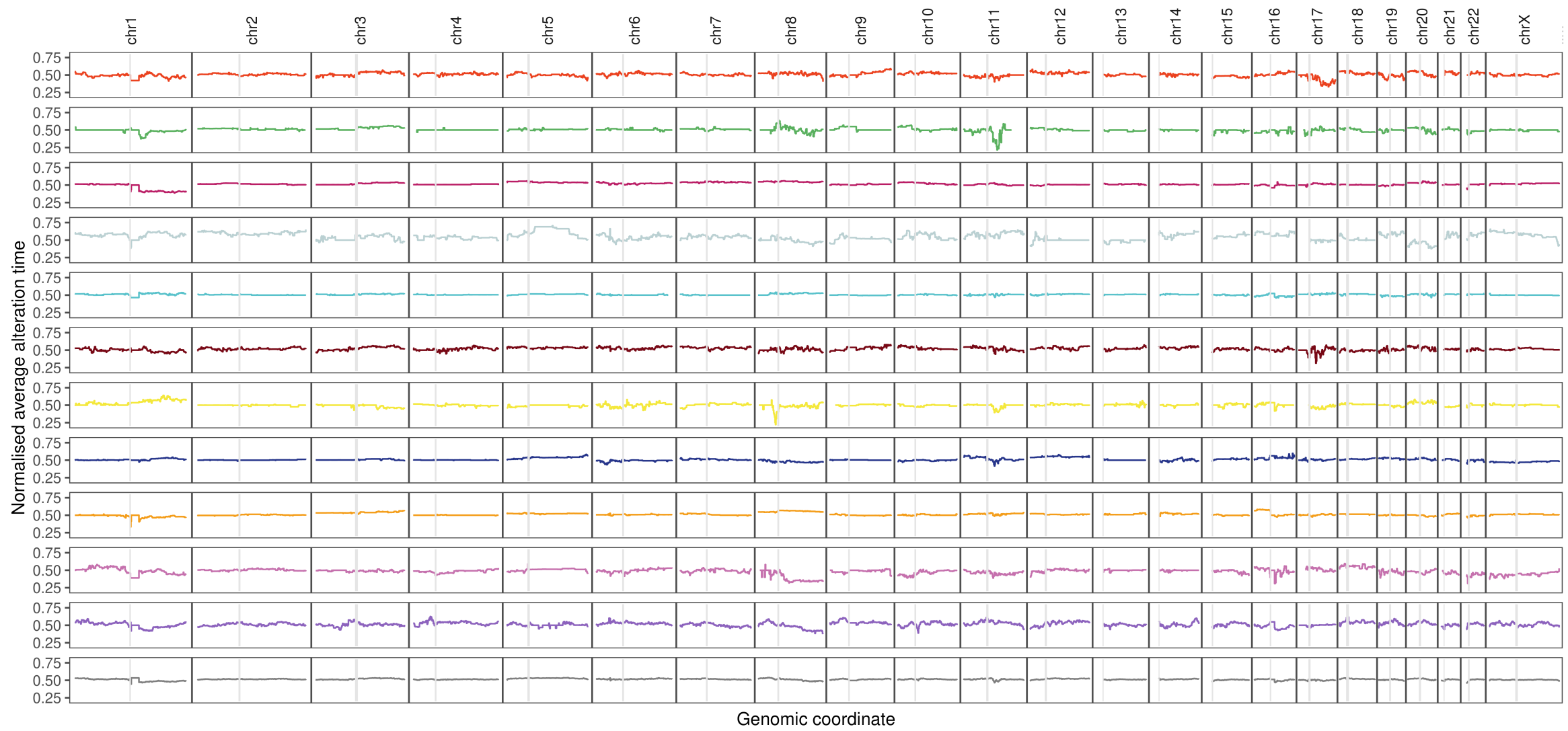

(a) Genomic subtypes

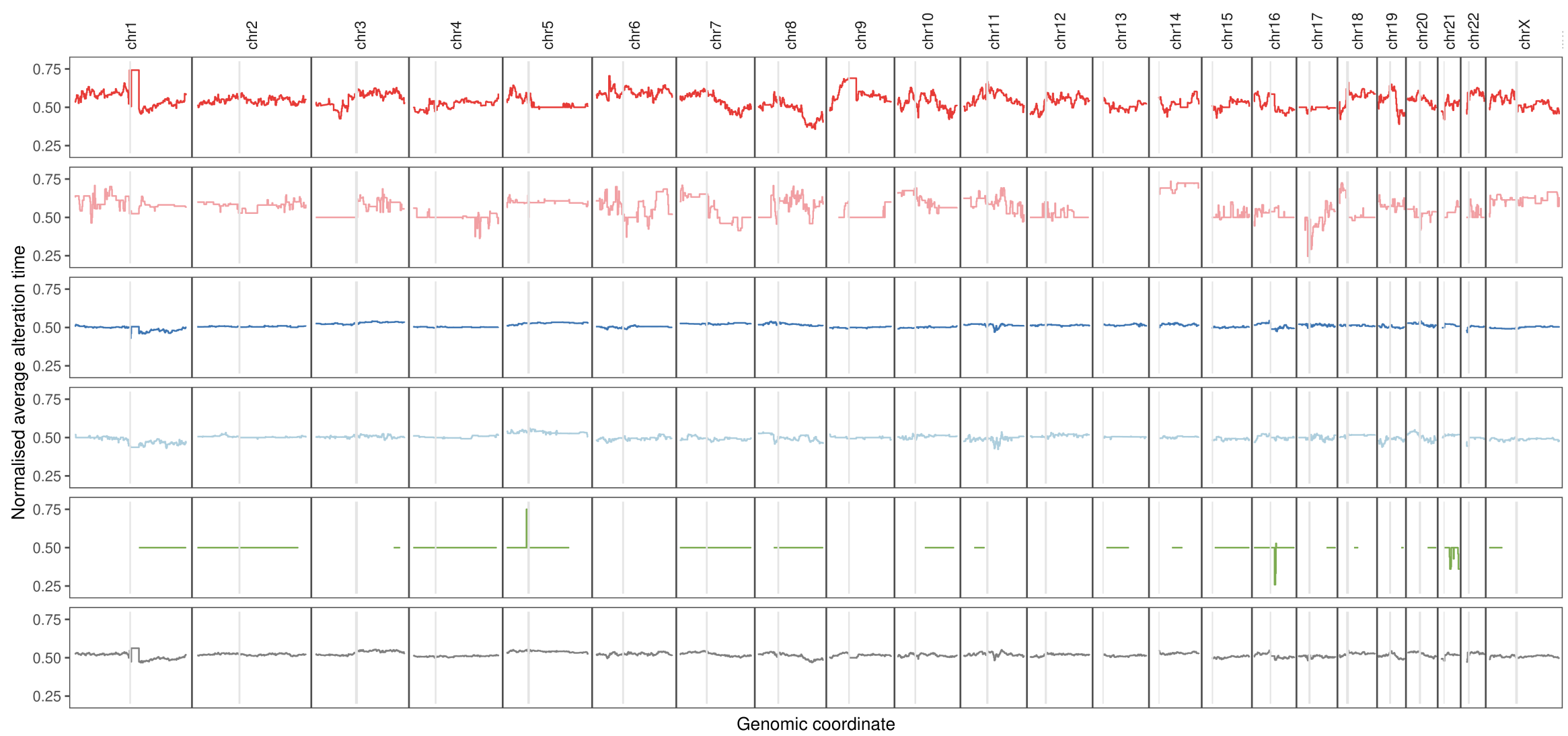

(b) PAM50 subtypes

Supplementary Figure 2: The timing of gains in primary tumours across subtypes. On the y-axis, smaller values denote earlier gains, while larger values denote later gains. Timing values have been averaged and normalised as described in the methods.

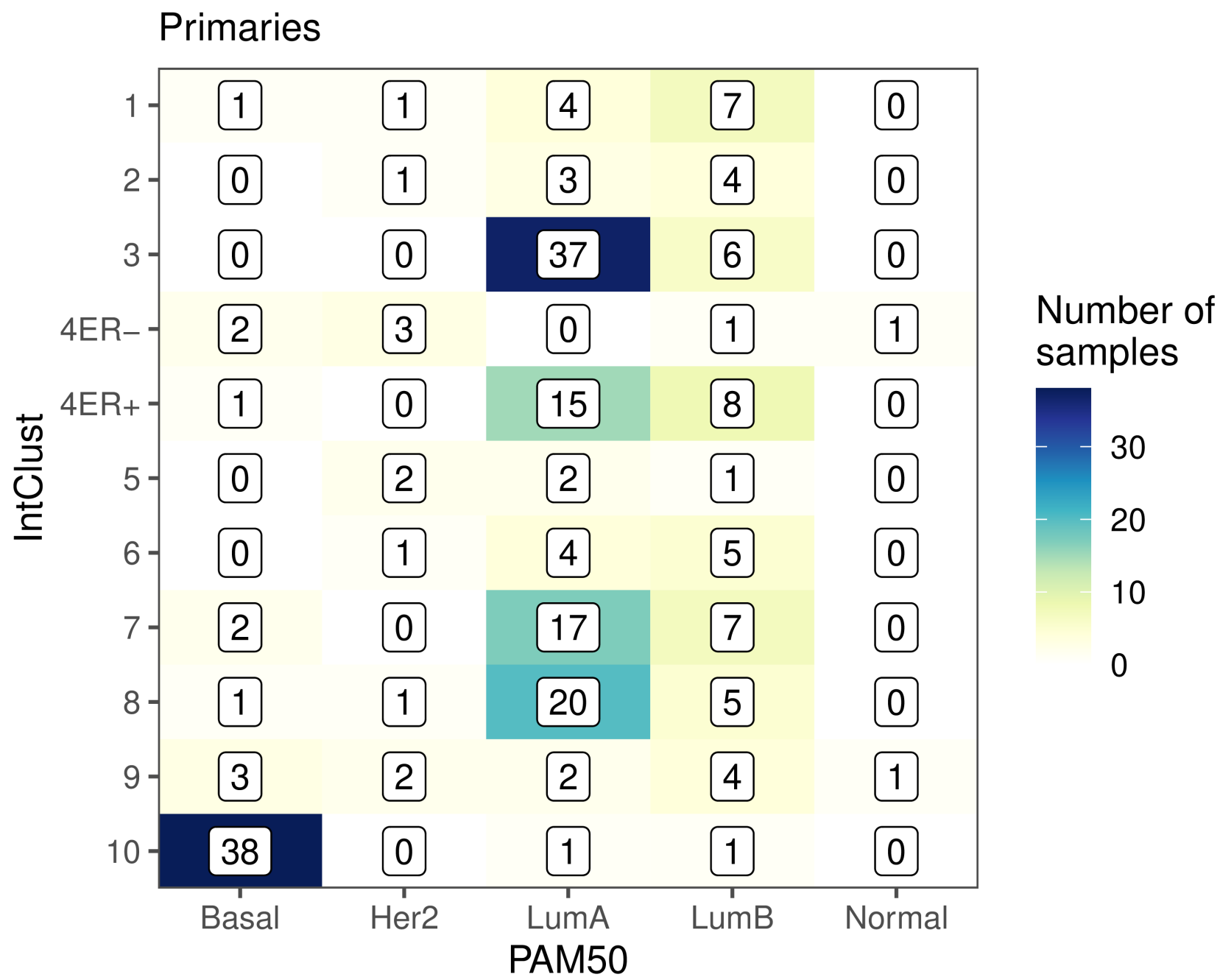

(a) Primaries

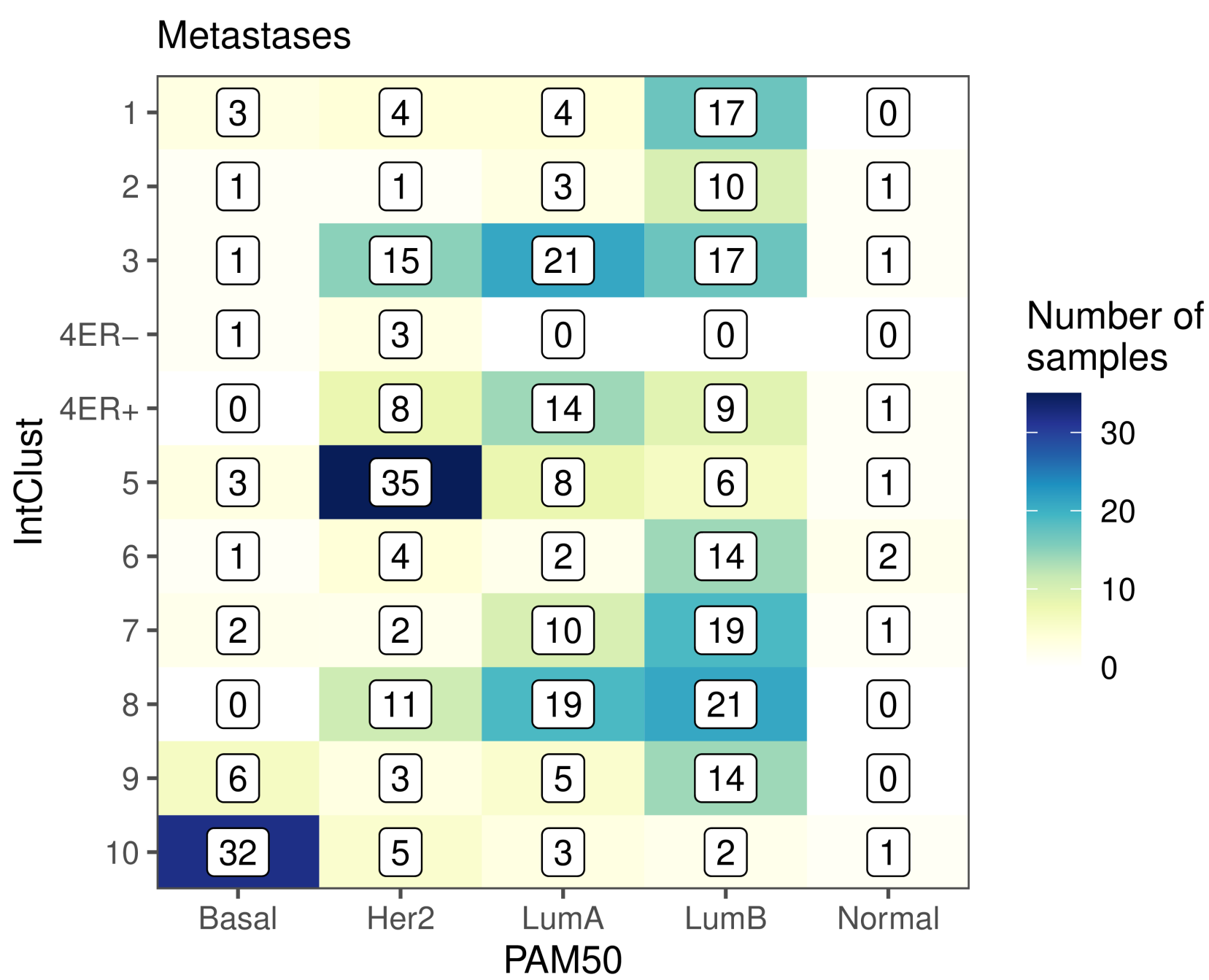

(b) Metastases

Supplementary Figure 3: The relationship between IntClust and PAM50 classifications in primary tumours and metastases. Tiles show the number of samples assigned to each pair of classifications.

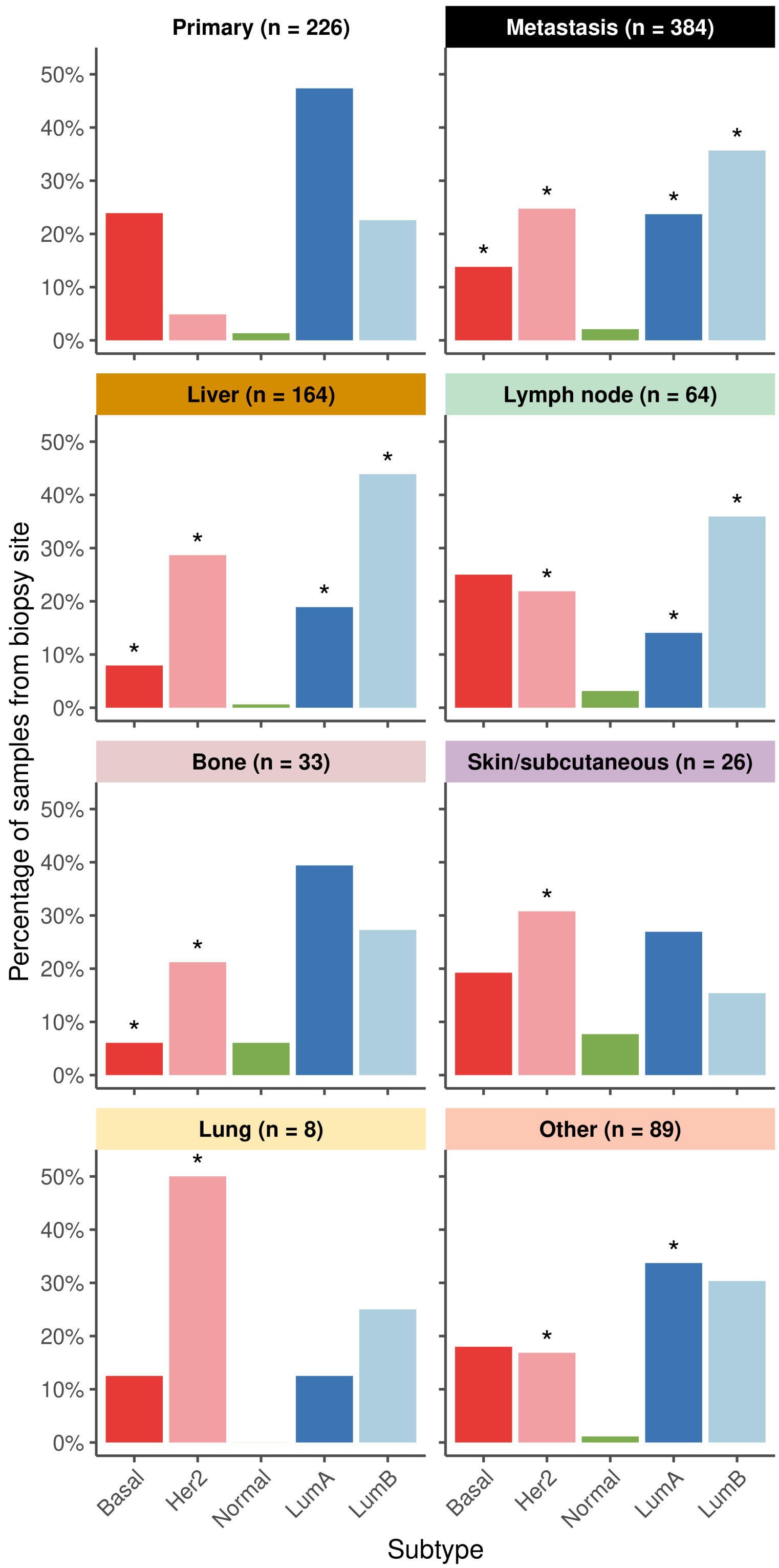

Supplementary Figure 4: The distribution of breast cancer samples belonging to each PAM50 subtype in primary breast tumours, breast cancer metastases, and at breast cancer metastatic sites. Asterisks denote where the frequency of a subtype in a metastatic site is significantly different to its frequency in primary breast tumours (binomial test, FDR-adjusted $p < 0.05$).

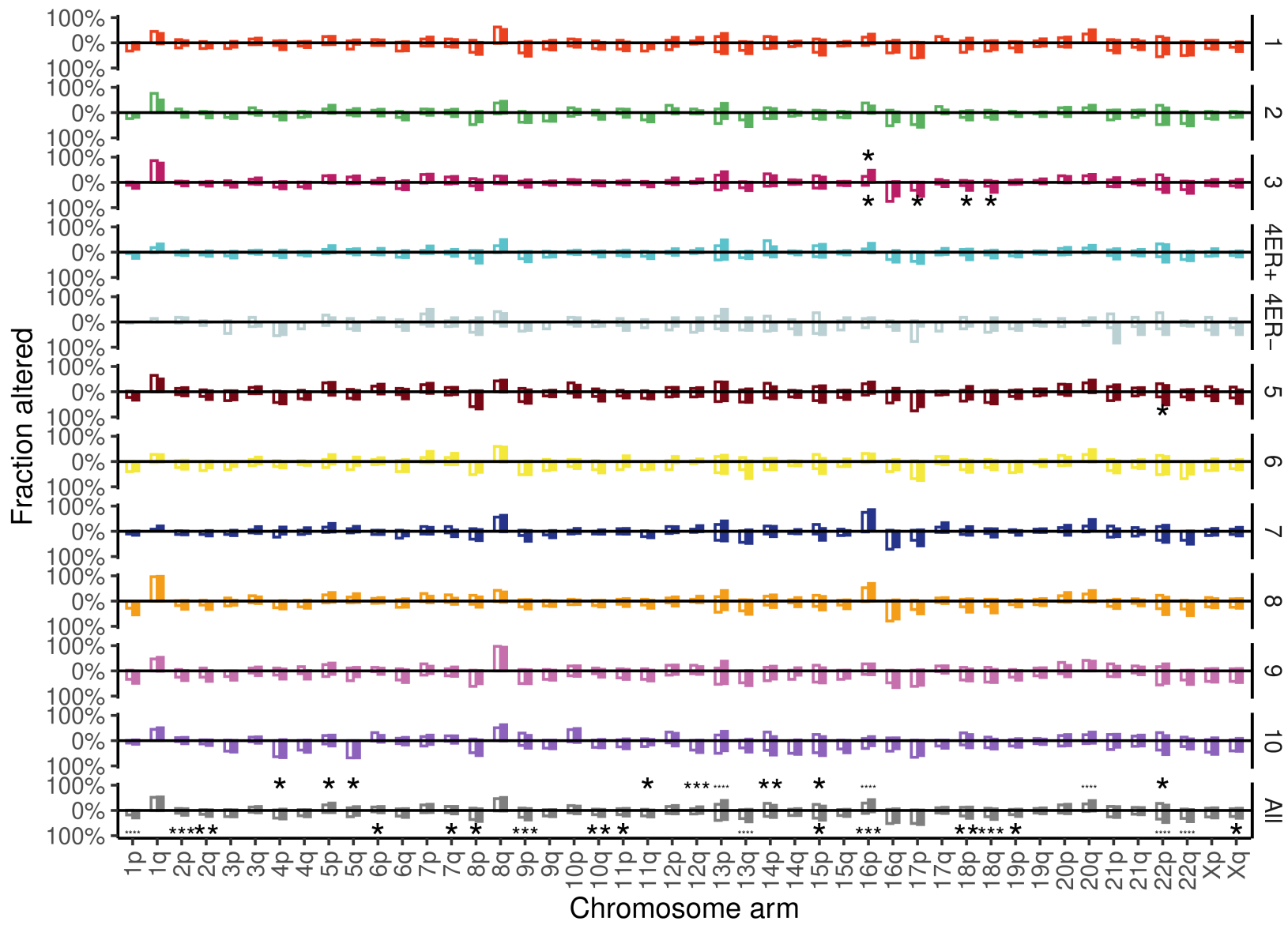

(a) Genomic subtypes

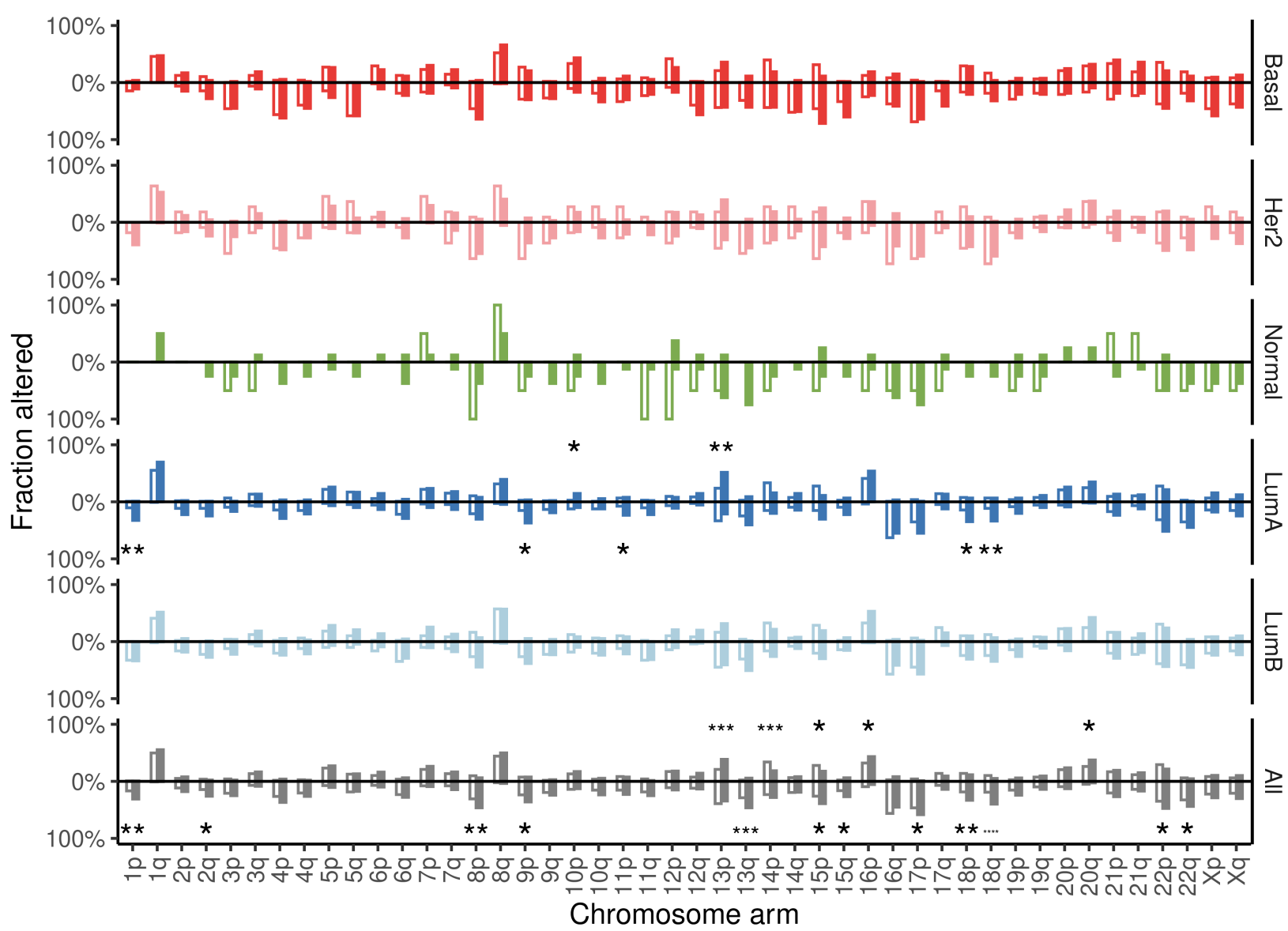

(b) PAM50 subtypes

Supplementary Figure 5: The frequency of arm-level aneuploidies across subtypes. Unfilled and filled boxes represent the frequency in primary tumours and metastases respectively. The frequency of gains and losses are shown above and below the zero line respectively. Asterisks denote arms where the frequency of aneuploidy was significantly different between primary tumours and metastases (Fisher test; FDR-adjusted; *: $p < 0.05$, **: $p < 0.01$, ***: $p < 0.001$, ****: $p < 0.0001$). Aneuploidy was defined as >80% of the arm length having integer copy number different from the rounded ploidy.

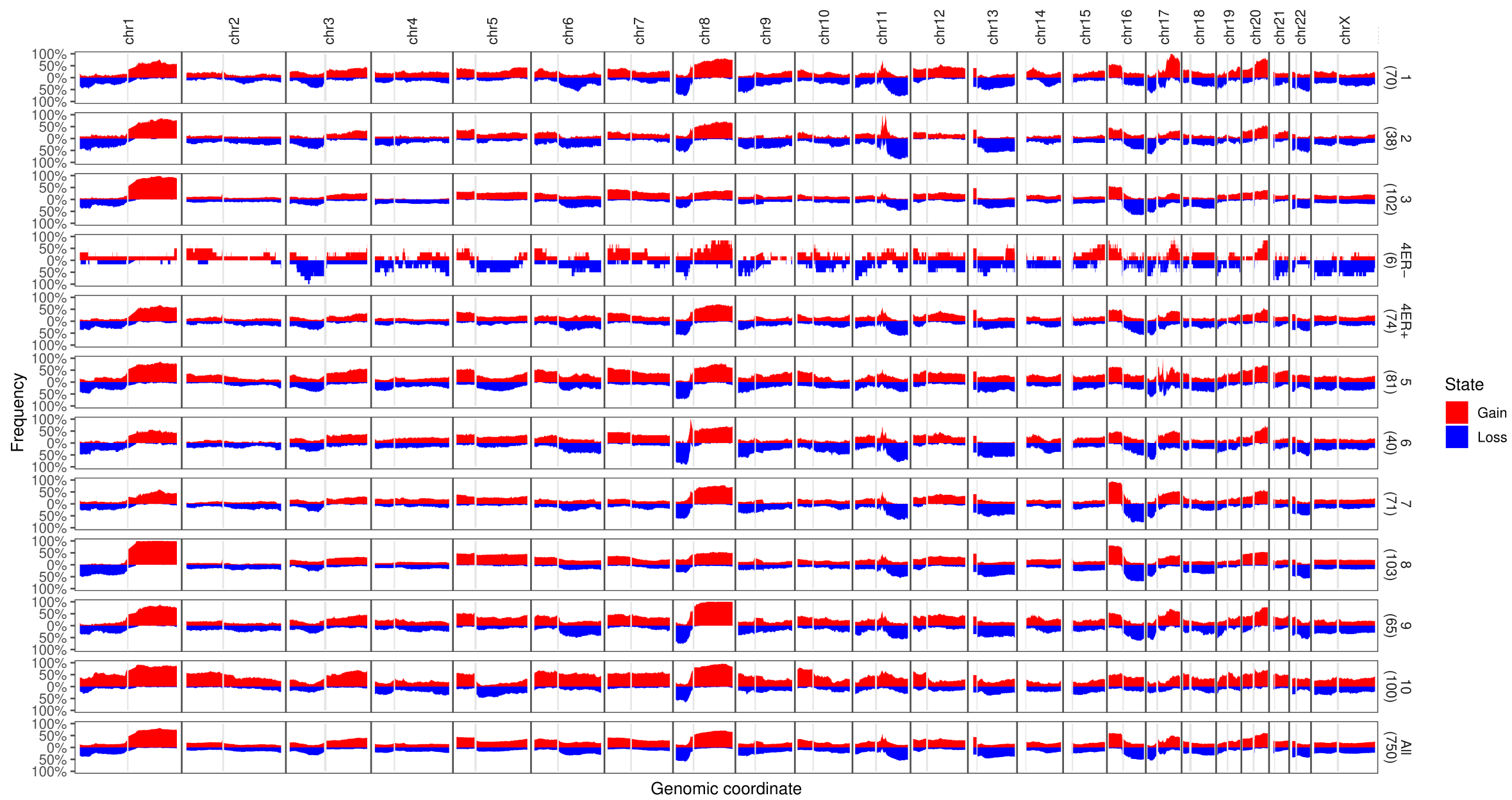

(a) Genomic subtypes

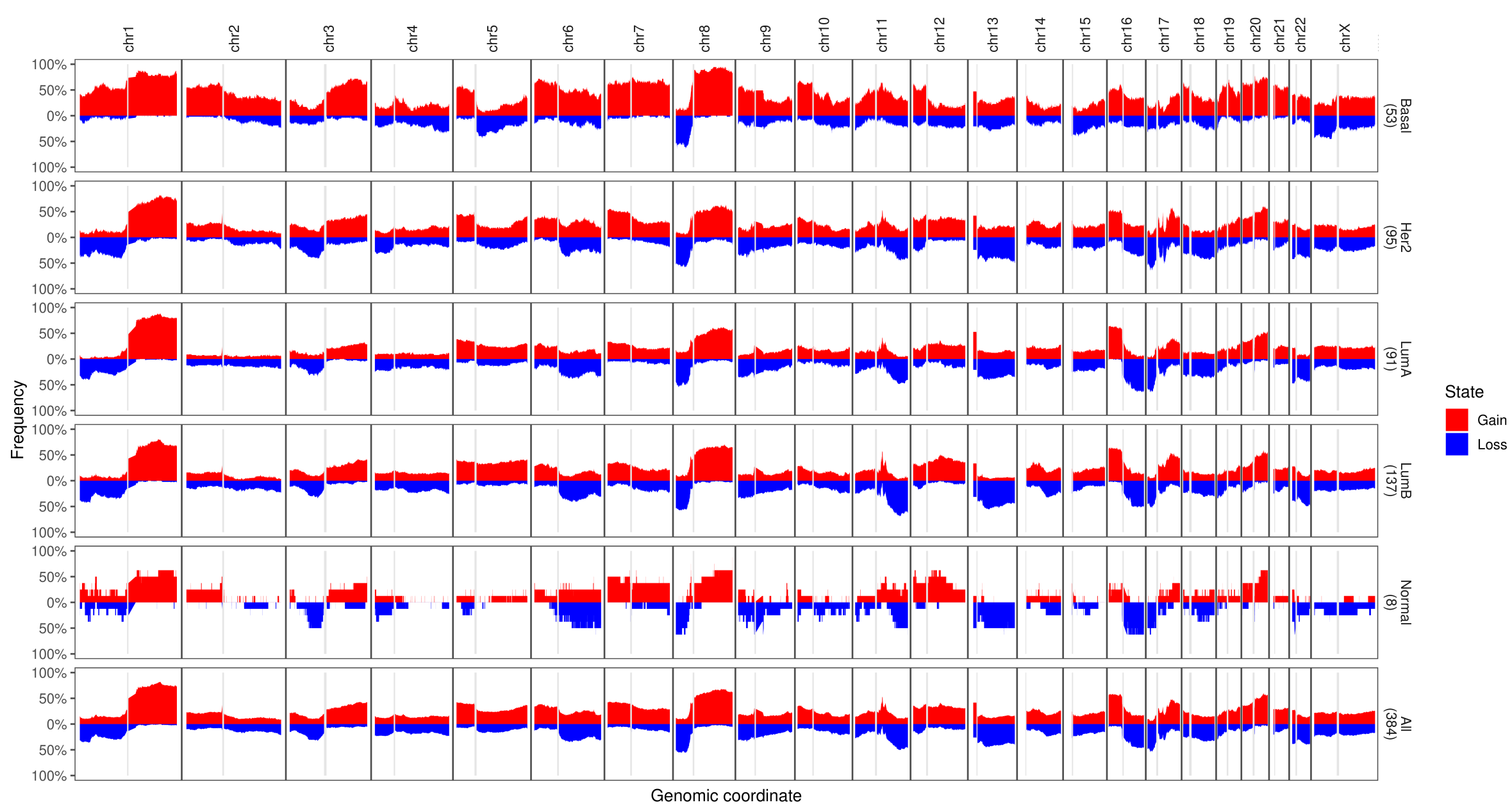

(b) PAM50 subtypes

Supplementary Figure 6: The frequency and genomic distribution of CNAs in metastases across subtypes. The frequency of gains is represented by the red shaded area above the zero line; the frequency of losses is represented by the blue shaded area below the zero line. Light grey boxes indicate the location of centromeres. The number of samples in each subtype is indicated in brackets.

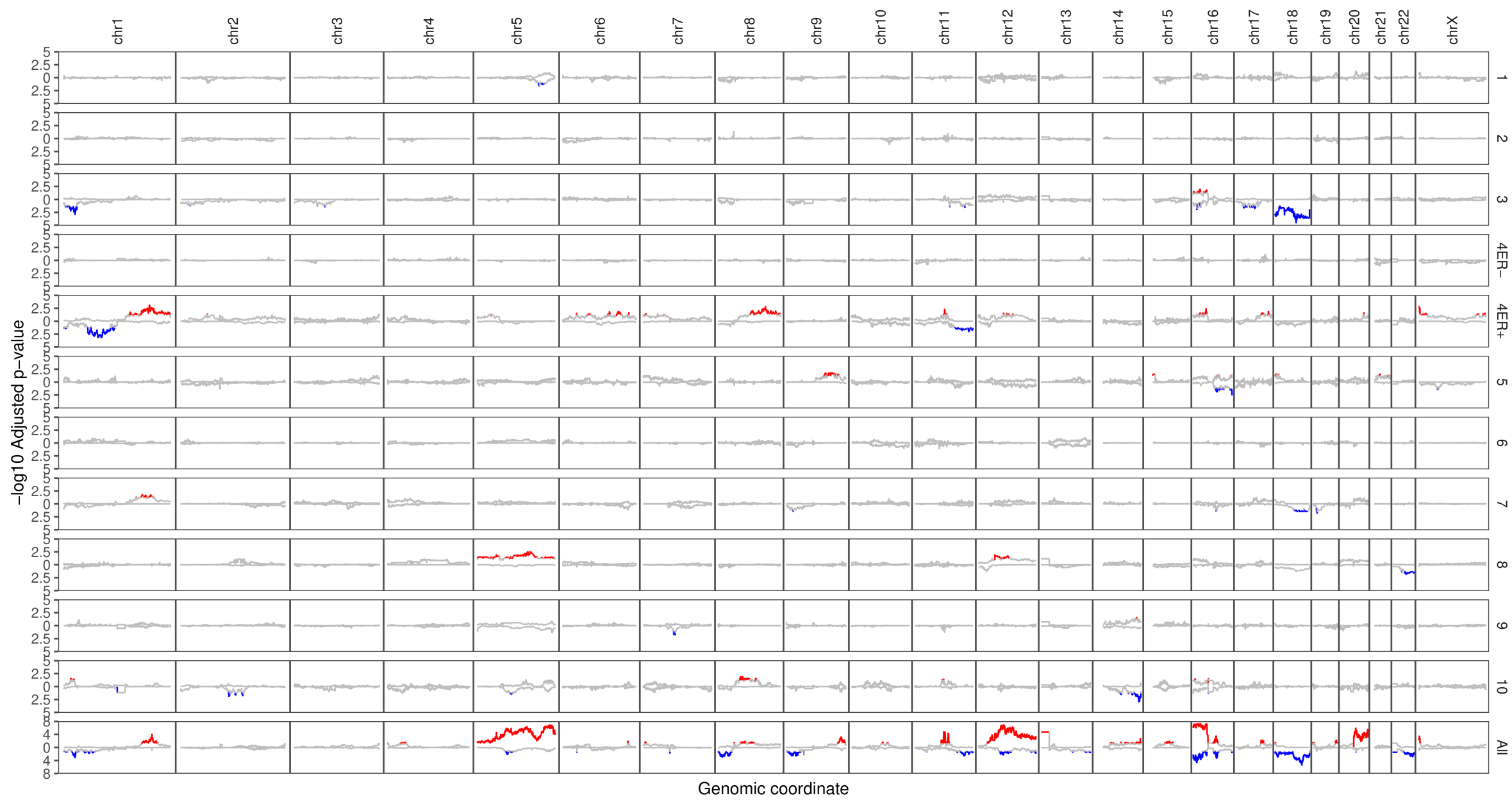

(a) Genomic subtypes

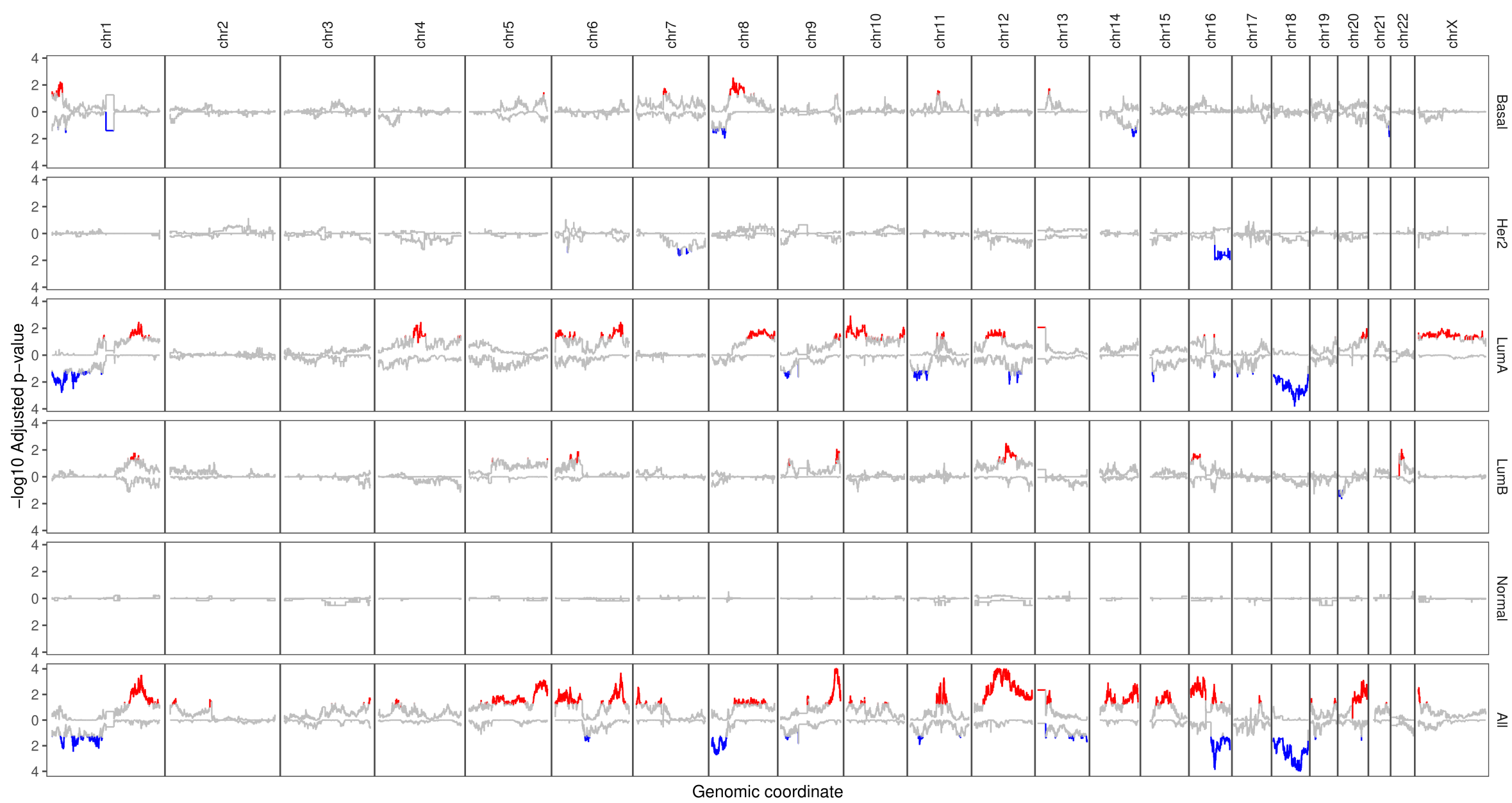

(b) PAM50 subtypes

Supplementary Figure 7: The statistical significance of differences in CNA frequency between primary tumours and metastases across the genome and subtypes. Line height represents the strength of evidence for a difference, with p-values for gains and losses shown above and below the zero line respectively. Coloured line segments indicate genomic regions where the CNA frequency differed significantly between primary tumours and metastases in either direction (two-sided chi-squared test; FDR-adjusted p-value < 0.05); red for gains, and blue for losses.

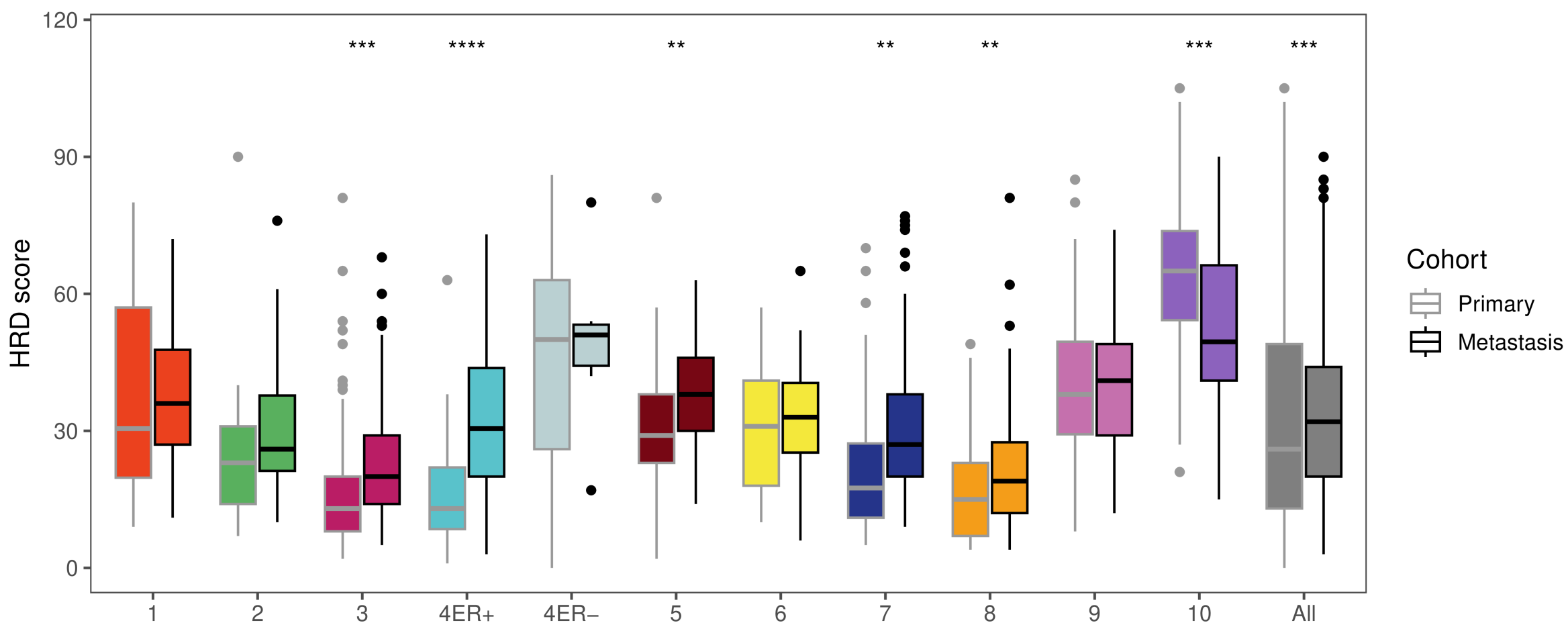

(a) Genomic subtypes

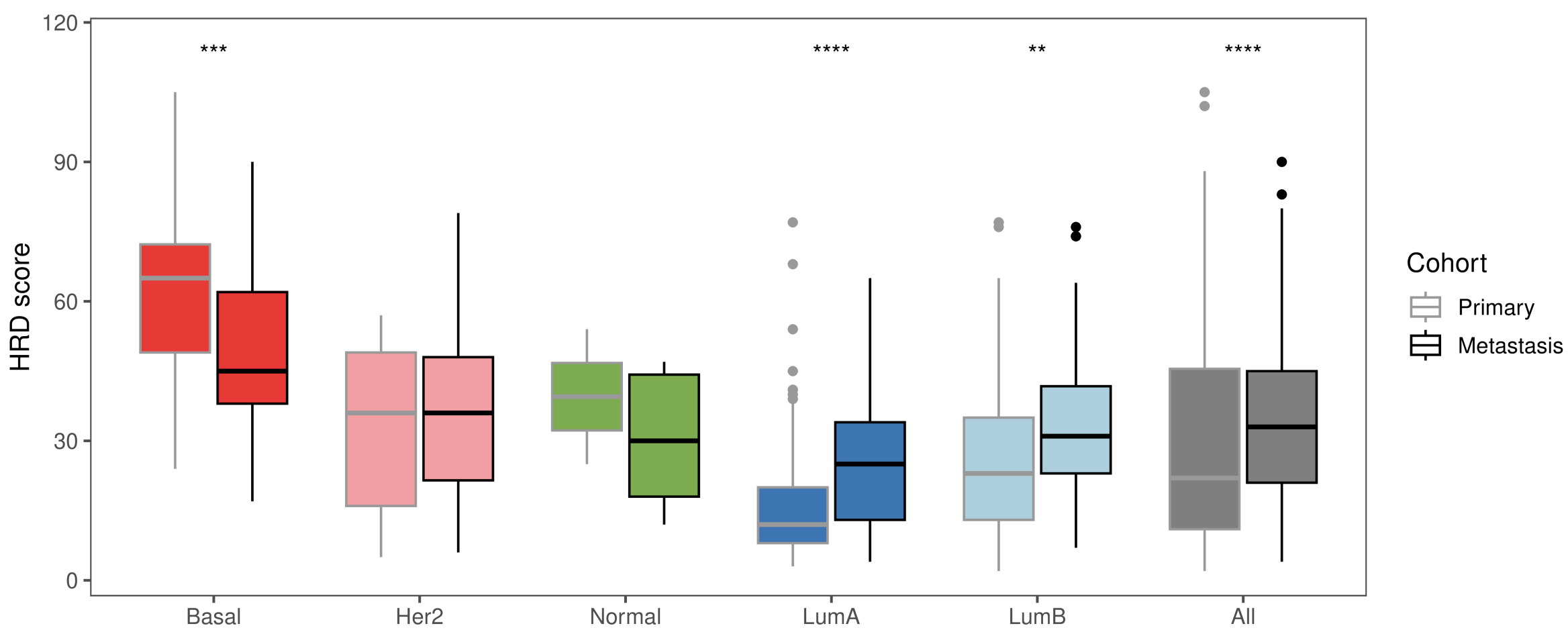

(b) PAM50 subtypes

Supplementary Figure 8: HRD scores in primary and metastatic cohorts across subtypes. HRD scores were calculated as described in the methods. Asterisks denote significant differences between primary tumours and metastases according to Mann-Whitney U tests (FDR-adjusted; *: $p < 0.05$, **: $p < 0.01$, ***: $p < 0.001$, ****: $p < 0.0001$)

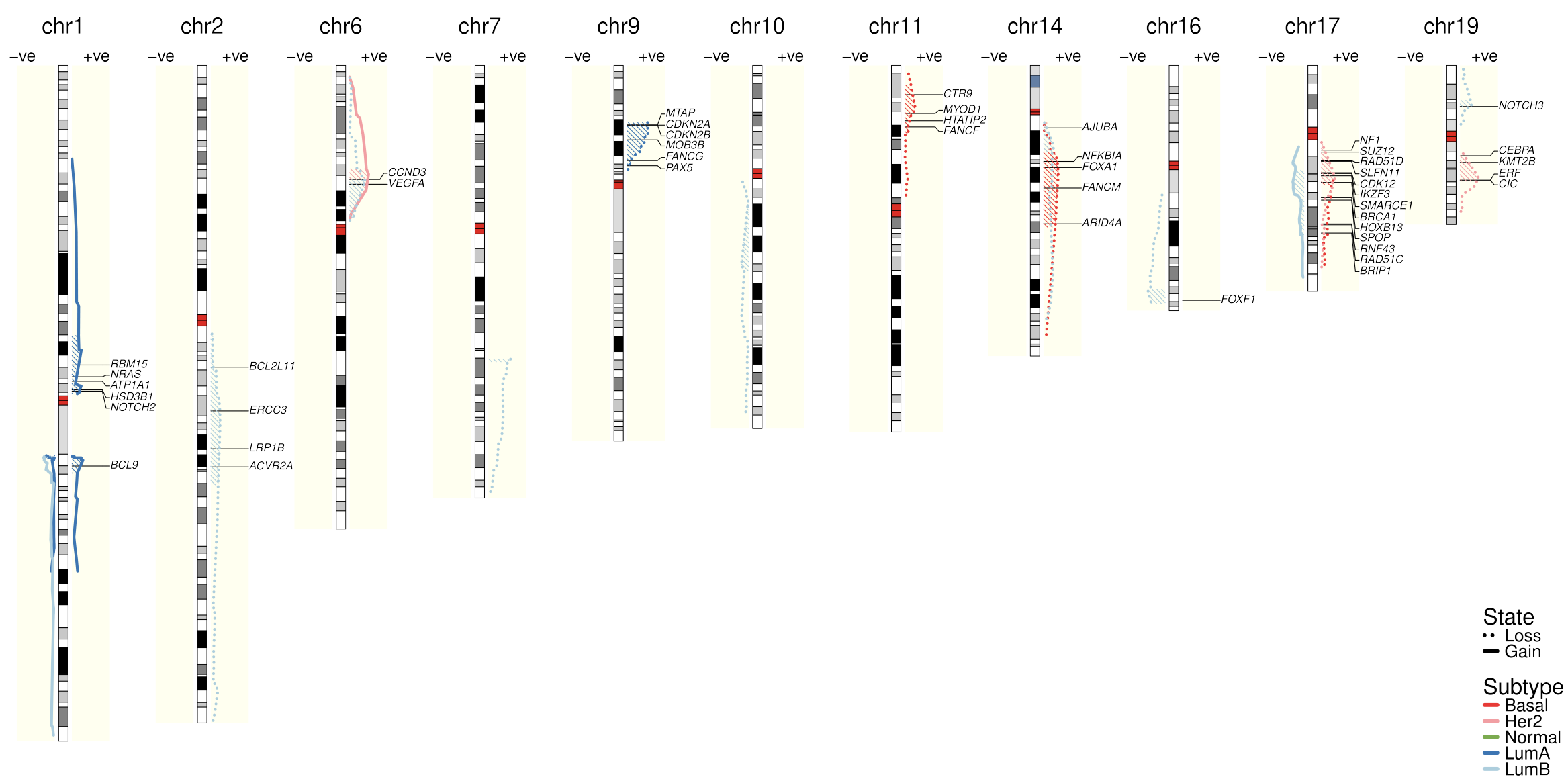

Supplementary Figure 9: Selection peaks detected with BISCUT breakpoint analysis. Lines' distance from each chromosome represents the distance between the breakpoint distributions of primary tumours and metastases. Lines to the left and right of chromosomes represent negative and positive selection respectively; solid and dashed lines represent selection of gains and losses respectively. Hashed areas indicate the location of significant peaks as calculated by BISCUT. The location of genes in the OncoKB database which fall within significant peaks are indicated.
